# Detecting differential growth of microbial populations with Gaussian process regression

**DOI:** 10.1101/gr.210286.116

**Published:** 2017-02

**Authors:** Peter D. Tonner, Cynthia L. Darnell, Barbara E. Engelhardt, Amy K. Schmid

**Affiliations:** 1 Program in Computational Biology and Bioinformatics, Duke University, Durham, North Carolina 27708, USA;; 2 Biology Department, Duke University, Durham, North Carolina 27708, USA;; 3 Computer Science Department, Center for Statistics and Machine Learning, Princeton University, Princeton, New Jersey 08540, USA

## Abstract

Microbial growth curves are used to study differential effects of media, genetics, and stress on microbial population growth. Consequently, many modeling frameworks exist to capture microbial population growth measurements. However, current models are designed to quantify growth under conditions for which growth has a specific functional form. Extensions to these models are required to quantify the effects of perturbations, which often exhibit nonstandard growth curves. Rather than assume specific functional forms for experimental perturbations, we developed a general and robust model of microbial population growth curves using Gaussian process (GP) regression. GP regression modeling of high-resolution time-series growth data enables accurate quantification of population growth and allows explicit control of effects from other covariates such as genetic background. This framework substantially outperforms commonly used microbial population growth models, particularly when modeling growth data from environmentally stressed populations. We apply the GP growth model and develop statistical tests to quantify the differential effects of environmental perturbations on microbial growth across a large compendium of genotypes in archaea and yeast. This method accurately identifies known transcriptional regulators and implicates novel regulators of growth under standard and stress conditions in the model archaeal organism *Halobacterium salinarum*. For yeast, our method correctly identifies known phenotypes for a diversity of genetic backgrounds under cyclohexamide stress and also detects previously unidentified oxidative stress sensitivity across a subset of strains. Together, these results demonstrate that the GP models are interpretable, recapitulating biological knowledge of growth response while providing new insights into the relevant parameters affecting microbial population growth.

Quantification and prediction of microbial growth is a central challenge relevant to industrial production of value-added chemicals, food safety, and microbe–environment interactions (McKellar and Lu 2003; Ross and Dalgaard 2003; Nichols et al. 2011; Lewis et al. 2012). Parametric models of microbial population growth assume a sigmoid growth function with three characteristic growth phases captured by three parameters: *lag phase time* (lag phase; λ), during which no growth occurs; *maximum growth rate during logarithmic growth* (log phase; μ_max_), a phase of rapid growth; and *asymptotic carrying capacity* (stationary phase; *A*), reached when nutrients are exhausted in stationary phase (Monod 1949; Zwietering et al. 1990; Baranyi and Roberts 1995; Egli 2009). Another quantification of growth is the area under the growth curve (AUC), also known as *growth potential* (Todor et al. 2014).

Microbial populations encounter shifts away from optimum growth conditions in their environment that require adaptation in order to survive. These shifts, generally referred to as *stress conditions*, include reactive oxygen species (ROS) accumulation, temperature variation, and osmotic fluctuation. These conditions chemically damage or denature macromolecules such as proteins, nucleic acids, and lipids, compromising cellular viability (Imlay 2003; Kühn and Klipp 2012; Verghese et al. 2012). During stress response, the cell state changes from a growth-centric to a survival-centric configuration in which the transcriptional and translational programs are redirected to regulate alternative pathways that repair damage and restore homeostasis (Lu et al. 2009). When stress is severe, the repair program becomes overwhelmed. In this case, the population growth rate observed by optical density (OD) decreases, plateaus, and may even become negative upon cell lysis.

Existing methods used to model and predict microbial population growth from time-series measurements are parametric functions known as *primary* or *secondary models* (McKellar and Lu 2003; Ross and Dalgaard 2003; Peleg and Corradini 2011). *Primary models* are used to fit data from a population growing on a single main nutrient source (e.g., sugar carbon source) and often assume a sigmoid growth function. This functional assumption leads to inaccurate fits for growth data that do not have a characteristic sigmoid growth function (Sekse et al. 2012; Palacios et al. 2014). *Secondary models* were developed to incorporate additional parameters affecting growth and to capture stress effects (Peleg and Corradini 2011). The significance of differential growth across conditions can be quantified through statistical hypothesis testing (Gommers et al. 1988). However, incorporating condition-specific deviations to the sigmoidal growth function requires a priori knowledge of how stress perturbations affect growth. For example, a common assumption is that population growth rate follows an Arrhenius equation in response to temperature changes (Barsa et al. 2012). As an alternative to parametric models of population growth, nonparametric models have been developed (di Sciascio and Amicarelli 2008; Cao et al. 2010; Palacios et al. 2014); however, many of these models still depend upon parametric primary models, parameters that require knowledge of the biological response to growth perturbations, or complicated fitting procedures of the nonparametric model, such as optimization of neural network weights (Palacios et al. 2014). Current models of microbial growth are therefore limited in their general application to novel microbial growth phenotypes.

Across all domains of life, stress response mechanisms at the level of gene transcription have been identified that regulate cellular protection and repair (Gasch et al. 2000; Bonneau et al. 2007; Fiebig et al. 2015). These regulatory programs are induced in response to stress conditions and protect cells exposed to one type of stress against other stressors (Jenkins et al. 1988; Lu et al. 2009). Conversely, cells also induce stress-specific responses to aid survival for a particular condition (Stephen et al. 1995; Zuber 2009). The hypersaline-adapted, or halophilic, archaeon *Halobacterium salinarum* is a model organism uniquely suited to study microbial stress response because it survives extremely high levels of ultraviolet (UV), ROS, heat shock, and other stressors in its desert salt lake niche (Ng et al. 2000; Oren 2008). As such, *H. salinarum* has been extensively studied as a model system for transcription regulatory network architecture and function in response to stress (Schmid et al. 2009, 2011; Todor et al. 2013, 2014; Tonner et al. 2015). A gene regulatory network inferred from transcriptomic data predicts that over 70 transcription factors (TFs) may regulate genes whose products adjust physiology and repair damage incurred by stress (Bonneau et al. 2007; Brooks et al. 2014). Network predictions have been used to characterize the full set of TF target genes and physiological roles of TFs that control the response to oxidative stress through RosR and AsnC (Sharma et al. 2012; Plaisier et al. 2014; Tonner et al. 2015), nutrient availability through TrmB (Schmid et al. 2009; Todor et al. 2013, 2014), metals through SirR (Kaur et al. 2006), iron homeostasis through Idr1 and Idr2 (Schmid et al. 2011), and copper response through VNG1179C (Kaur et al. 2006; Plaisier et al. 2014). Despite this knowledge, the cellular regulators of growth that respond to environmental perturbation remain understudied in *H. salinarum* and other archaeal species. In particular, the phenotypic impact of mutations to known TFs under alternate stress conditions—and the downstream effect of those mutations on the function of the global regulatory network—remains unclear for *H. salinarum* (Brooks et al. 2014) and many other understudied microorganisms (Yoon et al. 2011, 2013).

Here, we develop a Gaussian process (GP) regression model of microbial growth to overcome the limitations of parametric growth modeling. GPs are distributions on arbitrary functions, where any finite number of observations of the function are distributed as a multivariate normal (MVN) in a computationally tractable framework (Rasmussen and Williams 2006). Because GP regression fits an arbitrary functional form, it is able to model growth curves that deviate from a sigmoid form. We compare our model with several primary parametric models and establish the ability of GP regression to accurately model growth curves from *H. salinarum* under standard and stress treatments across genetic backgrounds. We show that the fitted GP model recovers biologically interpretable measures of microbial growth. We develop statistical tests of differential growth response between two experiments via data likelihoods computed from the fitted GP regression model. We call this model and testing framework *Bayesian Growth Rate Effect Analysis and Test* (B-GREAT). To demonstrate the general utility of B-GREAT, we applied it to yeast population growth data under diverse stress conditions and genetic backgrounds (Liti et al. 2009). In both *H. salinarum* and yeast applications, B-GREAT recapitulates known differential growth phenotypes and enables discovery of novel phenotypes.

## Results

**Figure 1.**
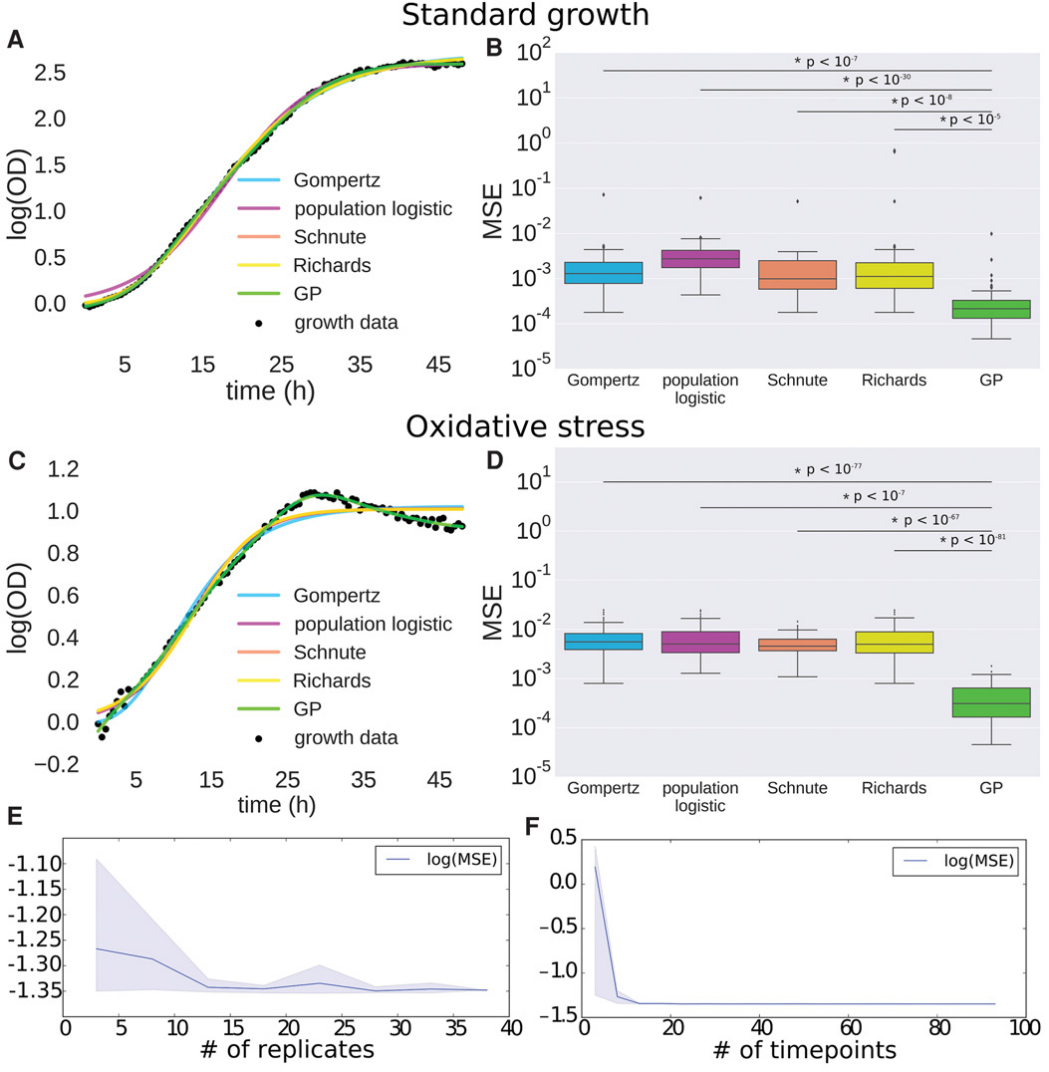
Gaussian process (GP) regression outperforms primary growth models. (*A*) Comparison of GP regression and primary growth models (Gompertz, population logistic, Schnute, and Richards) on microbial growth data under standard conditions. (*B*) Logarithm of mean squared error (MSE) for primary growth models compared with GP regression on microbial population growth under standard conditions. Bars with an asterisk indicate a significant difference between GP MSE and primary growth model MSE as determined by a one-sided *t*-test. *P*-values of the significance are indicated *above* the bars. (*C*) Comparison of GP regression and primary growth models on microbial growth data under oxidative stress. (*D*) Logarithm of MSE for primary growth models compared with GP regression on microbial population growth under oxidative stress. Bars with asterisks as in *B*. (*E*,*F*) Measure of MSE as a function of the number of replicates (*E*) and of time points (*F*) for GP regression. Solid lines represent mean MSE, and shaded regions represent empirical 90% confidence regions calculated from three random samplings of data at each number of replicates or time points.

We developed a GP regression model to capture population growth data and applied this model to data from seven *H. salinarum* TF mutants (Table 1). The growth of these strains was compared to the Δ*ura3* parent strain from which the mutants were derived under optimum nutrient conditions (referred to as “standard conditions”) and chronic oxidative stress (see Methods). OD, which quantifies cell density, was measured using a high-throughput plate reader (Fig. 1; Supplemental Fig. S1). Population growth phenotypes were measured in a minimum of 12 samples per mutant per condition, sampled every 30 min over 48 h for a total of 12,720 data points (Supplemental Table S1). Chronic oxidative stress was induced by the addition of 0.333 mM paraquat (PQ) when the culture was inoculated. The growth rate of these TF strains under standard conditions during log phase has been tested previously (Kaur et al. 2006; Schmid et al. 2009, 2011; Sharma et al. 2012; Plaisier et al. 2014), but only the growth rates of TF knockout mutants Δ*asnC*, Δ*trmB*, and Δ*rosR* have been tested under PQ conditions (Table 1; Schmid et al. 2009; Sharma et al. 2012; Plaisier et al. 2014).

**Table 1.**
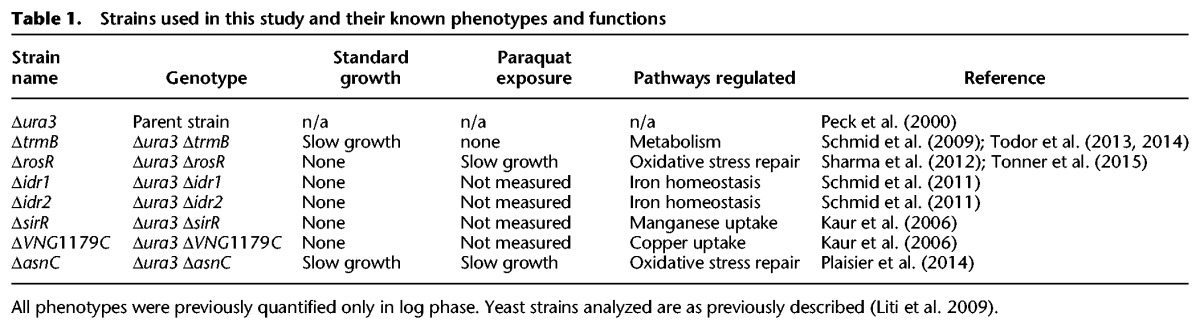
Strains used in this study and their known phenotypes and functions

### GP regression model of microbial population growth

In order to model the diverse growth phenotypes observed under both standard and oxidative stress conditions, a probabilistic model of population growth was constructed using GP regression (Fig. 1; Supplemental Fig. S1). GP regression is a Bayesian nonparametric model that describes the distribution over a function *f*(*x*), of which any finite number of observations {*x*, *f*(*x*)} have a MVN distribution (see Methods) (Rasmussen and Williams 2006). The GP model is described by its prior mean and covariance functions (μ(*x*) and κ(*x*,*x*′), respectively). In this study, prior mean μ(*x*) was set to zero, as is standard (Rasmussen and Williams 2006). The kernel function was set to a radial basis function (RBF), κ(*x*,*x*′), defining the covariance matrix of this MVN distribution.

GP regression places a prior on all arbitrary functions mapping time to OD, where the kernel function and parameterization encourage a specific smoothness of the function. Independent and identically distributed (IID) Gaussian noise with mean zero and variance σ^2^ is assumed in each function observation *y* = *f*(*x*) + *N*(0,σ^2^). Estimating the parameters of a GP regression model on microbial growth data is performed by maximizing the data likelihood with respect to the kernel function parameters (Rasmussen and Williams 2006). We refer to our model (and associated tests, described below) as *Bayesian Growth Rate Effect Analysis and Test* (B-GREAT).

### Evaluating kernel function choice for GP growth modeling

In order to ensure that our choice of RBF kernel function accurately represented the data, we tested the use of Matérn and linear kernel functions compared with the RBF kernel function. Matérn kernels are stationary, like RBF kernels, and model the covariance of data points as a function of their distance in *x*. Linear kernels are of the form $k(x,y)=\sum_{i=1}^{p}\sigma_{i}x_{i}y_{i},$ and the covariance increases with the magnitude of the covariates (Rasmussen and Williams 2006). The GP model with each of the three kernels was used to fit growth data for the Δ*ura3* parent strain under standard conditions. Model fits were assessed by the Bayesian information criterion (BIC) (Neath and Cavanaugh 2012). GPs with Matérn and RBF kernels have lower BIC scores than those with linear kernels, indicating that GP models with these kernels are more likely than those with a linear kernel (Supplemental Fig. S2; Neath and Cavanaugh 2012). From this, we conclude that our use of RBF kernel functions is sufficient for these data.

### B-GREAT outperforms primary growth models

B-GREAT was used to fit time-series growth data from *H. salinarum* Δ*ura3* parent strain populations under both standard and oxidative stress conditions. In order to benchmark GP regression as a model of microbial population growth, GP prediction error was compared to those from four primary growth models: Gompertz (Zwietering et al. 1990), population logistic regression (Zwietering et al. 1990), Schnute (1981), and Richards (1959; see Methods). All of these primary growth models depend on parameters λ and μ_max_, corresponding to lag time and maximum growth rate, respectively (Zwietering et al. 1990; Baranyi and Roberts 1995), of a sigmoidal growth curve. The Gompertz, logistic regression, and Richards models also include a parameter for carrying capacity (*A*). Both the Richards and Schnute models include parameters that modify the sigmoidal shape of the growth curve but do not have direct biological interpretations (Zwietering et al. 1990). The computational time to estimate classical growth parameters was somewhat smaller for primary growth models than for GP regression, but the difference in time is negligible to the researcher (Supplemental Fig. S3).

To test model accuracy of GP regression against primary growth models, data were randomly split into training and test sets including 80% and 20% of the data, respectively. We calculated mean squared error (MSE) between test data and model prediction given training data for each model under both standard conditions and oxidative stress. The fit to the data from all models was qualitatively (Fig. 1A) and quantitatively (Fig. 1B) accurate under standard conditions. However, chronic oxidative stress modified the growth trajectory of *H. salinarum* populations such that the data deviated from primary model assumptions (Fig. 1C), and MSE increased by an order of magnitude across all methods besides GP regression (Fig. 1D). GP regression MSE under both standard and stress conditions was significantly lower than MSE for each of the primary models (one-sided *t*-test, *P* ≤ 10^−5^) (Fig. 1B,D). Unlike primary models, the difference in MSE between the standard and stress conditions for GP regression was only 2.6% (one-sided *t*-test, *P* = 0.90). This shows that B-GREAT models growth data from populations grown under standard and stress conditions with equivalent accuracy.

We next tested the accuracy of GP regression as a function of sampling density, both in the number of observed time points and the number of experimental replicates. We found that GP regression accuracy, measured using MSE, was relatively stable as sampling density decreases, and error did not increase until fewer than 12 replicates or eight time points were used for training (Fig. 1E,F). The maximum difference in MSE as a function of replicate number was 10.5%, while the maximum error as a function of time points was nearly five times higher with eight time points than with the original 96 time points. The increase in error as a function of a decrease in replicate number was gradual, while the error as a function of time points had a sharp inflection point when fewer than eight time points were used. Generally speaking, these error estimates are useful to guide experimental design for time-series growth data.

### GP regression recovers parameters of primary growth models

**Figure 2.**
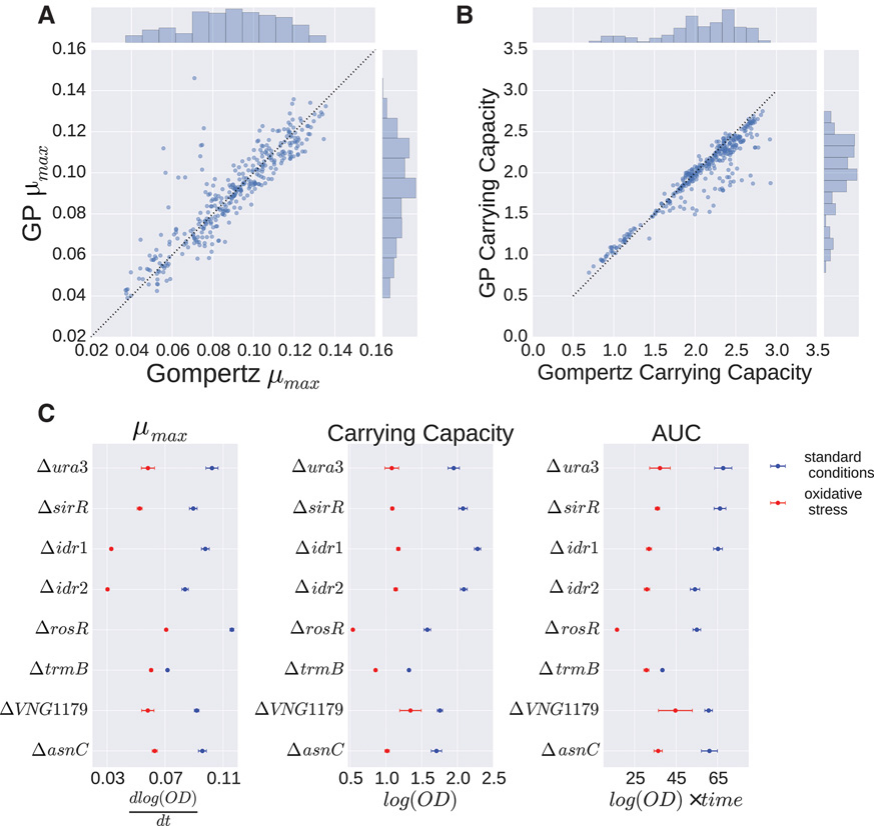
Growth parameters estimated using GP regression. (*A*,*B*) Correlation of parameter estimates of μ_max_ (*A*) and carrying capacity *A* (*B*) between Gompertz and GP regression. Dotted line represents the line *y* = *x*. (*C*) Posterior representations of growth parameters μ_max_, carrying capacity, and AUC are shown for each strain under standard conditions (blue) and oxidative stress (red). Points represent posterior mean; error bars, 95% credible regions.

To enable a biological interpretation of GP growth curves and a quantitative comparison with primary parametric model output, growth parameters of primary models—*A*, μ_max_, and AUC—were extracted from fitted GP models (see Methods). GP estimates of these parameters under standard growth conditions for the Δ*ura3* parent strain were well correlated with those from Gompertz regression (*r*^2^ = 0.903 for μ_max_ and *r*^2^ = 0.947 for *A*, *P* ≤ 10^−5^, Pearson correlation) (Fig. 2A,B). Estimates of *A* from Gompertz regression were slightly higher than those from GP regression for a subset of samples (Fig. 2B; Supplemental Fig. S4A). Conversely, estimates of μ_max_ from GP were higher than those from Gompertz for three growth curves due to instrument noise in the first few time points (Supplemental Fig. S4B). Despite these exceptions, the correlation in parameters was high across models.

GP regression estimates of *A*, μ_max_, and AUC for the Δ*ura3* parent strain were then compared with parameter estimates from seven TF deletion strains under both standard and oxidative stress conditions. According to these parameters, some mutant strains differed from the Δ*ura3* parent under standard conditions, while others differed under oxidative stress. For example, μ_max_ for the Δ*trmB* strain, a known nutrient responsive regulator, was lower than μ_max_ for the Δ*ura3* strain under standard conditions as expected from previous studies (Schmid et al. 2009; Todor et al. 2013, 2014). Estimates of *A* and AUC for the Δ*rosR* strain were lower than *A* and AUC for the other strains. We found significant differences for one or more parameters estimated from the Gompertz model between Δ*ura3* and TF mutant strains, except for Δ*asnC* under PQ stress (*t*-test, *P* ≤ 0.01; family-wise error rate [FWER] ≤ 0.25) (Supplemental Figs. S5, S6; Supplemental Table S2). Under both standard and oxidative stress conditions, all strains were considered significant for at least one growth parameter (Supplemental Figs. S5, S6). For *A*, Δ*sirR* was the only strain that was not significant under both conditions (Supplemental Figs. S5, S6). These results demonstrate that growth parameters estimated from GP models are biologically relevant and comparable to those estimated using primary models under standard conditions. GP has the added benefit of estimating these parameters accurately for stress conditions, although the biological interpretation may differ from parameters estimated for standard conditions.

### B-GREAT identifies known and novel differential growth phenotypes under standard conditions

**Figure 3.**
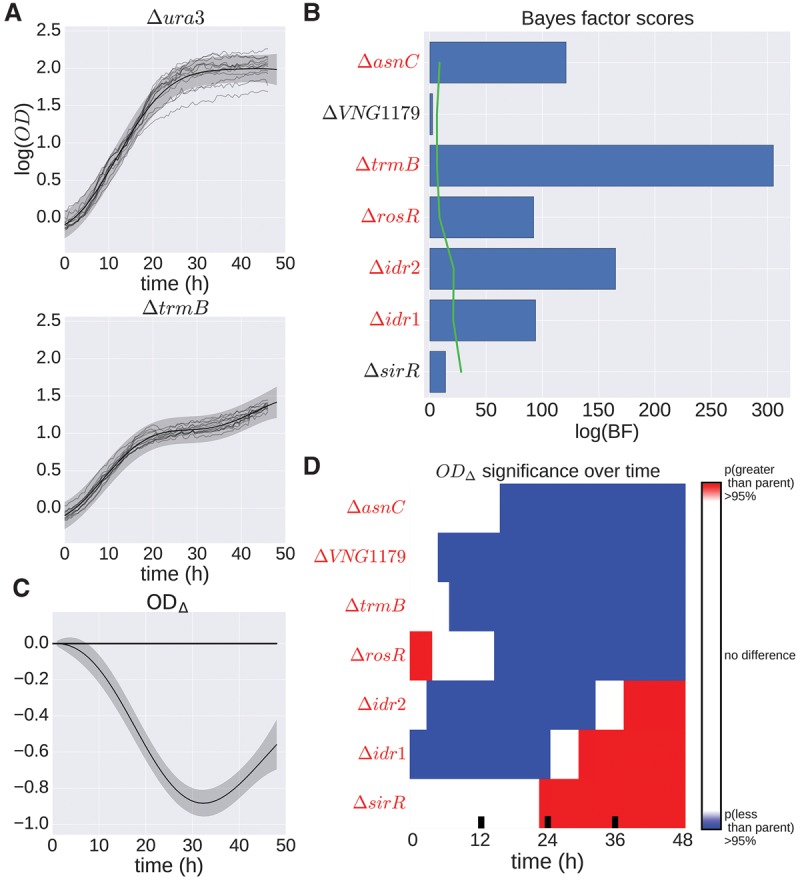
*H. salinarum* mutants with significant growth phenotypes under standard conditions. (*A*) Population growth data and GP model fit of *H. salinarum* parent strain Δ*ura3* (*top*) and Δ*trmB* (*bottom*) under standard growth. Light gray curves represent growth samples of each strain in different wells. Solid black lines and shaded gray regions indicate mean and 95% credible region of the GP model fit to the growth data, respectively. A single GP model was fit (equation 16) and separate growth predictions made for Δ*ura3* and Δ*trmB* (see Methods). (*B*) Bayes factors (BFs) for each mutant strain are shown as blue bars. Permuted BF scores representing an FDR ≤ 20% is indicated by the green line. Strains with a BF score with FDR ≤ 20% are in red italics. (*C*) The difference in growth level between Δ*trmB* and Δ*ura3* using the prediction of growth from the GP model. The solid line indicates mean difference, and the shaded region is the 95% credible region. Regions where the 95% credible region does not include zero suggest that the growth between the two strains is different at that time point with high probability. (*D*) Predicted difference between mutant and parent strain population growth using posterior function distributions as in the previous panel. Red and blue regions indicate a >95% probability that the mutant population growth is either higher or lower than the parent strain, respectively. Strains with OD_Δ_ 95% credible region not including zero at any time point are in red italics.

We next sought to identify differential growth phenotypes of TF mutants versus the Δ*ura3* parent strain under standard conditions. Testing for differences in growth phenotypes across strains using classical growth model parameters was difficult: (1) A separate test was conducted for each parameter; (2) comparing variation between multiple parameters was not straightforward because of differences in magnitude (Fig. 2C); and (3) *t*-tests of classical growth parameters were overly sensitive, calling nearly all strains significant for multiple parameters across conditions (Supplemental Figs. S5, S6). To overcome these limitations, we developed a statistical test using Bayes factors (BFs) based on our GP regression model. B-GREAT was designed to capture differences across the entire time series, irrespective of the magnitude and shape of the deviation. Specifically, B-GREAT compares the data likelihood under two models, the null and alternative models. For the sake of efficiency, point estimates of the GP regression hyperparameters are computed instead of integrating over their uncertainty, making our BF estimates approximate (Kass and Raftery 1995; Stephens and Balding 2009). For the null model, *H*_0_, we used *f*(time), which indicates that the population growth under the condition of interest is the same between parent and mutant strain. For the alternative model, *H*_*A*_, we used OD(time, strain) = *f*(time, strain), which represents the function of the OD at a given time and for a specific strain, where a strain value of zero or one indicates parent strain or mutant strain, respectively. The covariate *strain* was added to the model by extending the RBF kernel of the GP to an additional input dimension (Rasmussen and Williams 2006). The alternative model assumes that a given mutant population has a different growth response phenotype than the parent strain while sharing some characteristic shape through the *time* covariate (Fig. 3A). Typically, larger BFs indicate evidence for the alternative hypothesis, suggesting differential growth across the covariate (Kass and Raftery 1995).

In order to compute the statistical significance for our test of differential growth, we used permutations to calibrate the false-discovery rate (FDR) of our BFs (Stephens and Balding 2009). To do this, we developed a permutation framework to quantify the distribution of the test statistic under a null hypothesis (see Methods). Specifically, across growth data for both parent and mutant strains, the label of strain background was randomly permuted for each time point. Values were permuted so as to maintain the underlying distribution of strain labels present in the original data. We performed 100 permutations that represent an empirical null distribution for each BF test, and BF scores corresponding to FDR ≤ 20% were considered significant (Stephens and Balding 2009). By using an estimate of the distribution of the test statistic under the null hypothesis, we quantified the FDR for a given test statistic threshold (Mangravite et al. 2013). We performed calibration via permutation in lieu of using a test statistic that has an approximate χ^2^ distribution for more precise calibration at the cost of additional computation (Fusi and Listgarten 2016).

BF scores calculated from B-GREAT fits on growth curves for each mutant strain represent the overall effect of the strain background on population growth. B-GREAT found that five of the seven TF mutants had significant BFs under standard growth conditions, meaning that the mutant strain showed differential growth compared with the parent strain (FDR ≤ 20%), including Δ*asnC*, Δ*trmB*, Δ*rosR*, Δ*idr2*, and Δ*idr1* (Fig. 3B). To gain further biological insight into the phenotypes of the five strains with differential growth, we developed a second metric, OD_Δ_, that quantifies the difference in parent and mutant strain population growth at each time point (see Methods, equation 20) (Supplemental Fig. S7; Benavoli and Mangili 2015). This difference is computed using the posterior estimates of parent and mutant strain growth of the fitted B-GREAT model. As we are interested in differences in the actual growth of strain populations and not in differences arising from noise in growth measurements, OD_Δ_ is computed using posterior estimates of the underlying growth function without local Gaussian noise. Specifically, we computed the probability of the mutant strain growth conditioning on the parent strain growth at each observation time point according to the MVN distribution. We thresholded this probability at 95% to quantify a growth difference between parent and mutant strain at each time point.

As in previous work (Schmid et al. 2009), OD_Δ_ showed that Δ*trmB* grows more slowly than the Δ*ura3* parent strain throughout the time course (Fig. 3C,D). In contrast, Δ*idr1* and Δ*idr2* grow more slowly than the parent strain during exponential phase but reach higher cell densities during the latter portion of the growth curve (Fig. 3D). Δ*rosR* exhibits the opposite growth pattern. The fifth strain with a novel differential growth phenotype, Δ*asnC*, is impaired for growth throughout the time course. Although the growth of Δ*idr1*, Δ*idr2*, Δ*rosR*, and Δ*asnC* strains has been studied during log phase under standard growth conditions previously (Schmid et al. 2011; Sharma et al. 2012; Plaisier et al. 2014), these represent novel stationary phase phenotypes. Taken together, these results demonstrate that B-GREAT and the OD_Δ_ metric provide a simple, biologically interpretable test of significance of differential growth that captures the complexity of growth phenotypes.

### Identification of differential growth phenotypes in response to oxidative stress

**Figure 4.**
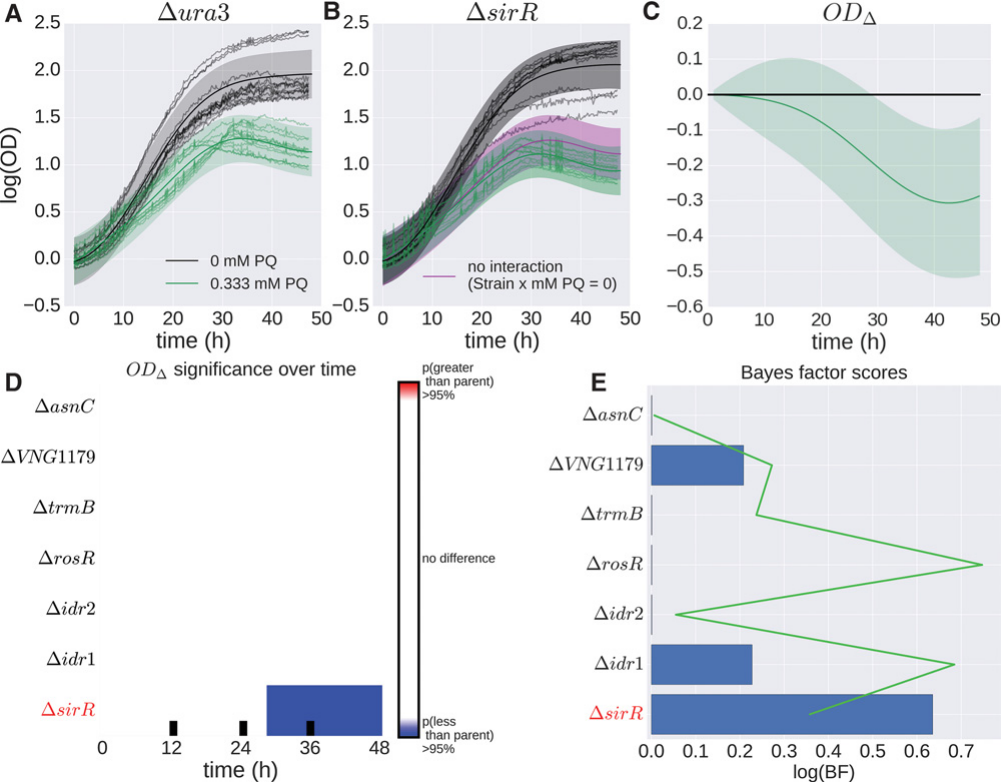
*H. salinarum* mutants with significant growth phenotypes under oxidative stress. (*A*,*B*) Example of population growth data from *H. salinarum* for mutant strain Δ*ura3* (*A*) and Δ*sirR* (*B*) under standard conditions (black) and chronic oxidative stress (green). Each curve represents a different sample of an experimental condition. Gaussian process predictions for these conditions are shown as a solid line (mean) and shaded region (variance). The purple line represents the growth prediction when the Strain × mM PQ interaction term is zero. (*C*) Difference computed between the mutant growth level with interaction term (Strain × mM PQ = 1) and mutant growth without interaction (Strain × mM PQ = 0); solid lines represent mean, and shaded regions indicate 95% credible regions. (*D*) Functional difference and permuted BF scores for mutant strains in response to oxidative stress. Functional difference is computed between mutant strain with and without an interaction term between mutant and stress condition. (*E*) BF score and permuted BFs for each strain are shown, where blue bars and green line represent observed BF and FDR ≤ 20% threshold, respectively. Strains with FDR ≤ 20% are in red italics.

We next used B-GREAT to quantify the change in population growth of the TF mutants and Δ*ura3* under chronic oxidative stress. The previous model of growth, *f*(time, strain), was extended to include an effect of PQ and an interaction term between strain and PQ stress: *f*(time, strain, mM PQ, (mM PQ × strain)) (equation 17). Here, mM PQ ∈ {0,1} represents the presence or absence of oxidative stress in the culture (Fig. 4A,B, green curves). The interaction term mM PQ × strain ∈ {0,1} is equal to one only for the mutant strain under oxidative stress, and to zero otherwise, and was included to test for differential growth of each mutant strain specific to oxidative stress (Fig. 4B, purple and green curves). The BF for this condition calculates the relative likelihood of the data with or without the interaction term mM PQ × strain (alternative and null models, respectively). This test statistic quantifies differential strain growth under oxidative stress while controlling for differences in growth between parent and mutant strain under standard conditions. The OD_Δ_ test was computed as the difference between mutant strain growth with or without the interaction term mM PQ × strain (Fig. 4C).

This extended B-GREAT framework detected significantly reduced growth relative to the parent strain for Δ*sirR* during the later stages of the time series under oxidative stress (OD_Δ_ 95% CI; BF FDR ≤ 20%) (Fig. 4C–E). Δ*sirR* was previously implicated in regulating genes involved in metal ion uptake (Kaur et al. 2006), but not in oxidative stress. No other strains were determined to have a significant growth impairment or improvement under PQ stress when differences in strain growth under standard conditions were controlled for in the model (Supplemental Fig. S8). These results indicate that it is straightforward to extend B-GREAT to control for known differential conditions to enable the discovery of novel differential growth phenotypes for previously characterized TF mutant strains.

### Meta-analysis improves differential growth phenotype detection

The strain Δ*rosR* is a known oxidative stress regulator that has previously been shown to regulate oxidative stress under both PQ and hydrogen peroxide exposure (Sharma et al. 2012; Tonner et al. 2015). Surprisingly, this strain did not exhibit a significant differential growth phenotype versus the parent strain under oxidative stress in our study (Fig. 4D,E). In order to determine the source of this discrepancy, we compared the growth data for Δ*rosR* generated for this study to data from a previous study (Supplemental Fig. S9; Sharma et al. 2012). We observed that Δ*ura3* reached a higher cell density in stationary phase than Δ*rosR* under standard conditions, showing a significant BF score (FDR ≤ 20%) in our study (Fig. 3B). Thus, controlling for the differential growth of the strain under standard conditions removed the differential stress condition phenotype. This difference during stationary phase under standard conditions was observed but not quantified in the previous study because only log phase was considered (Sharma et al. 2012).

**Figure 5.**
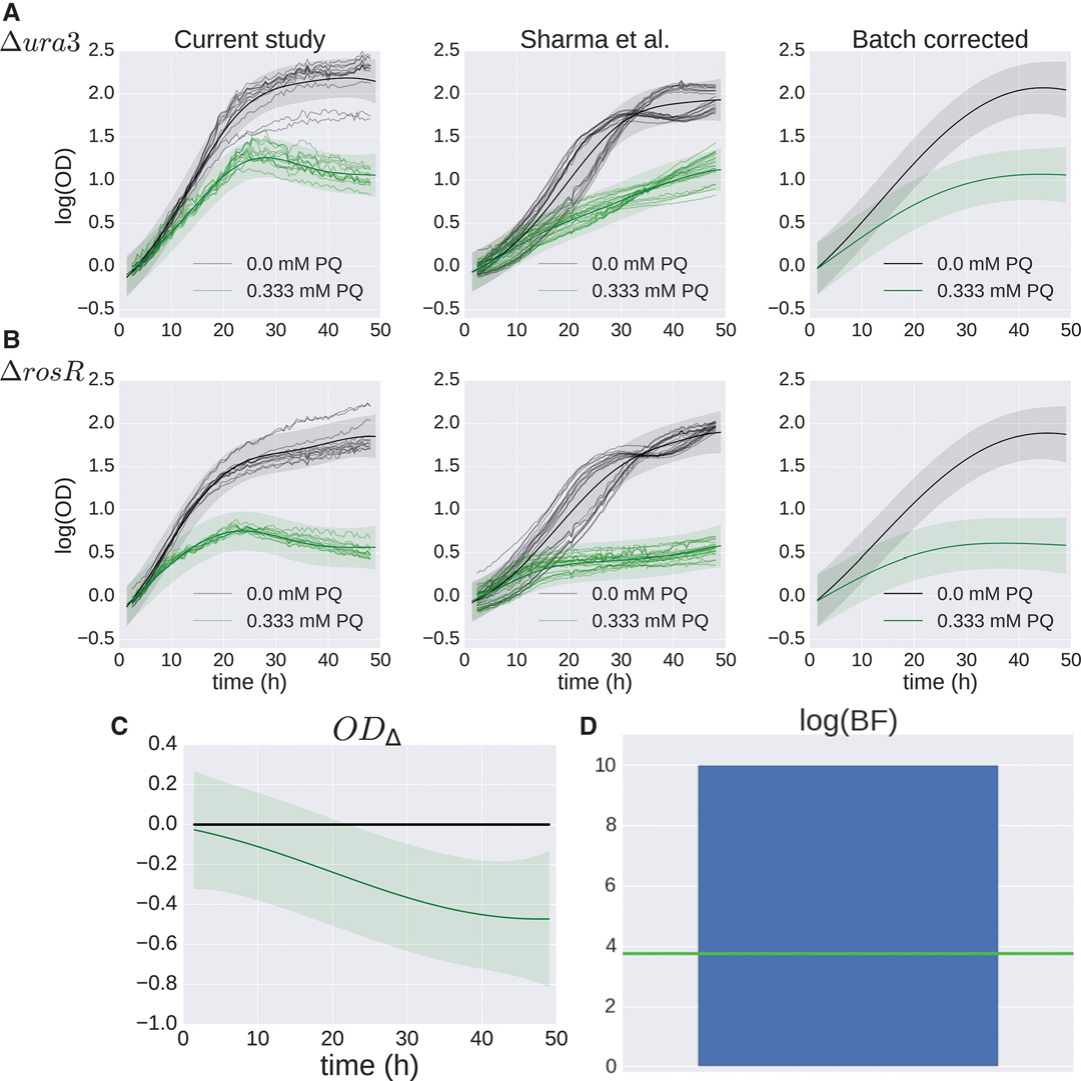
B-GREAT model of Δ*rosR* growth in response to oxidative stress across multiple studies. (*A*,*B*) Δ*ura3* (*A*) and Δ*rosR* (*B*) growth data under standard conditions (black) and oxidative stress (green). Individual samples from this study (*left*) and previously published data (*center*) (Sharma et al. 2012) are shown as shaded lines. The B-GREAT model prediction for each condition is shown as solid line and shaded region for mean and 95% credible region, respectively. The growth prediction for the underlying growth function estimated across studies is shown in the *right* column. (*C*) The difference between Δ*rosR* and Δ*ura3* growth for the underlying growth function corrected for batch effects, which shows an increased susceptibility of Δ*rosR* to oxidative stress relative to the parent strain. (*D*) log(BF) compared with permuted scores from the null distribution. Blue bar and green line represent observed BF and FDR ≤ 20% threshold, respectively.

To combine data from this study and from the previous study, we built a hierarchical GP model of growth that corrects for differences arising between batches of experiments (see Methods) (Hensman et al. 2013). Under this model, a shared growth function *g*(·) is estimated using a GP whose covariates match those in equation 17. Then systematic variation between the two studies was modeled as two GPs *f*_1_ and *f*_2_, whose means are given by the shared growth function *g*(·). Under this design, *g* represents the true growth phenotype of Δ*rosR* when corrected for study effects, and *f*_1_ and *f*_2_ represent the growth phenotype with study-specific effects included (Fig. 5A,B). From this model, we calculated the difference in Δ*rosR* growth with and without the (mM PQ × strain) interaction term. Once the variation between studies was corrected for, OD_Δ_ indicates that Δ*rosR* has a significant growth defect under oxidative stress (Fig. 5C), and the significance of this defect is confirmed with the BF score (FDR ≤ 20%) (Fig. 5D). This differential phenotype is consistent with the known function of the RosR TF as a genome-wide regulator of gene expression in response to oxidative stress (Tonner et al. 2015). These results demonstrate that this hierarchical model effectively combines cross-study data and corrects for study-specific effects, recapitulating the known phenotype of Δ*rosR* under PQ stress (Fig. 5C,D).

### B-GREAT identifies significant growth phenotypes across strains of yeast

**Figure 6.**
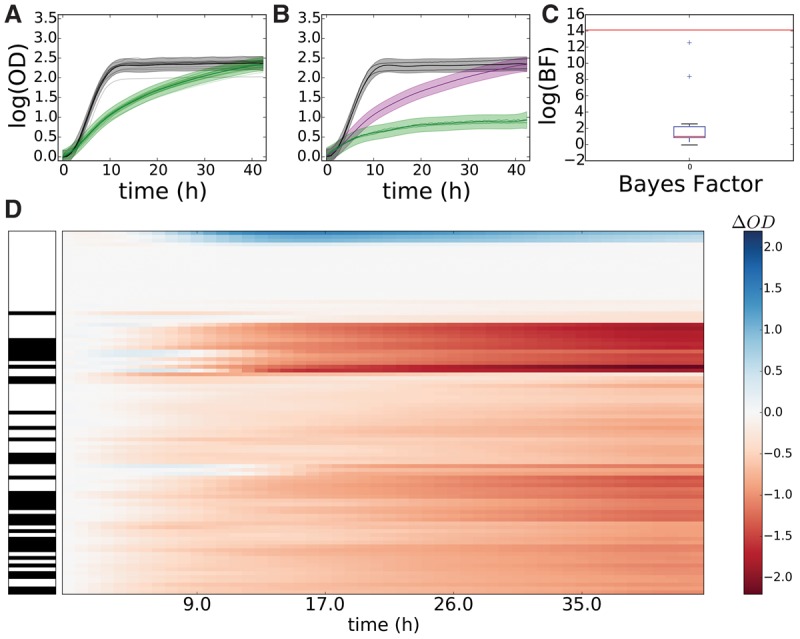
B-GREAT identifies significant growth phenotypes in yeast strains in response to paraquat. (*A*,*B*) Control strain BY4741 (*A*) and *Saccharomyces paradoxus* strain G4650 (*B*) growth under standard conditions (black) and under paraquat stress (green). Solid lines represent experimental data, and shaded regions represent B-GREAT model predictions. Purple-shaded region represents 95% credible region of B-GREAT prediction of G4650 in the absence of stress interaction (strain × stress = 0). (*C*) log(BF) (red line) and permuted log(BF)s (boxplot) of G4650 under paraquat stress according to B-GREAT. (*D*) OD_Δ_ scores of all yeast strains under paraquat exposure. *Left* column corresponds to *S. cerevisiae* (white) or *S. paradoxus* (black) strains. *Center* column represents magnitude of calculated OD_Δ_ over time for each strain.

To test the efficacy of B-GREAT as a general microbial population growth model, we applied our method to a large compendium of yeast growth profiles in which 96 domesticated and wild strains of *Saccharomyces cerevisiae* and *Saccharomyces paradoxus* were grown in various stress conditions (Liti et al. 2009). We used B-GREAT to test for differential growth between the control strain, BY4741, and all other strains under PQ stress and cycloheximide stress (Supplemental Figs. S10 and S11, respectively). Under both conditions, strains identified as having significant differential growth (FDR ≤ 20%) by B-GREAT were significantly overrepresented by *S. paradoxus* strains relative to *S. cerevisiae* (*P* ≤ 0.05, hypergeometric test). While *S. paradoxus* strains make up a minority of the strains in the data (37.5%), they constituted the majority of the strains with differential growth in response to PQ (65%, *P* ≤ 2.3 × 10^−7^) and cycloheximide (79.3%, *P* ≤ 1.8 × 10^−9^) (Fig. 6; Supplemental Figs. S10, S11). The increased resistance of *S. paradoxus* strains to cycloheximide relative to that of *S. cerevisiae* strains had been previously detected (Liti et al. 2009). However, in our B-GREAT analysis, we also detected significantly decreased resistance of *S. paradoxus* to PQ, a novel finding.

The yeast strain with the highest BF score, *S. paradoxus* G4650, showed severely inhibited growth in response to PQ stress compared with the BY4741 control (Fig. 6A–C). G4650 was isolated from fossilized guano in Italy, with no previously reported sensitivity to oxidative stress (Liti et al. 2009). When we extended the analysis to all strains in the data set, we saw a trend for *S. paradoxus* strains to grow poorly under PQ exposure compared with the BY4741 control (Fig. 6D). Together, these results show that B-GREAT recapitulates known biology and identifies new differential phenotypes from previous studies on large collections of strains with diverse genetic backgrounds.

## Discussion

In this study, we developed B-GREAT, a general model of microbial population growth using GP regression. B-GREAT overcomes the limitations of primary parametric models and enables discovery of novel growth phenotypes for genetically and environmentally perturbed microbial populations. We showed that B-GREAT is equivalently accurate under nonstandard conditions (Fig. 1B); moreover, its model accuracy is resilient to decreases in observation sampling (Fig. 1E,F). GP regression can recover growth statistics of log phase (μ_max_) and stationary phase (carrying capacity, *A*), enabling direct comparison of these variables to results from primary growth models (Fig. 2). Our comparisons demonstrated that GP regression outperforms primary parametric growth models in capturing growth, both under standard conditions and under nonstandard stress conditions (Fig. 1).

In our results, we highlighted important properties of the B-GREAT method for modeling microbial growth. GP models allow the inference of smooth underlying growth functions through the length-scale parameter, explicitly accounting for experimental noise. This is in contrast to other recent models such as linear spline fitting in generalized additive models (GAMs), which are sensitive to technical variation (Sekse et al. 2012), or polynomial splines in the *grofit* package, which require cross-validation to estimate parameters (Kahm et al. 2010). Given the large proportion of cellular machinery whose production correlates linearly with growth rate (Pedersen et al. 1978; You et al. 2013), differentiating general growth impairments from specific, stress-related impairments is important for biological interpretation of model fits. GP regression enables this process by easily incorporating multiple dimensions through the addition of length-scale parameters for each covariate, modeling and controlling for an arbitrary number of covariates (Fig. 4). For example, the number of strains with differential effects increases from one to five out of seven if the mM PQ × strain interaction term is removed (Fig. 4D; Supplemental Fig. S12), demonstrating the importance of this covariate. Additionally, GP regression may be extended to model data in which experimental variance is more complicated than simple independent Gaussian noise (Fig. 5; Shah et al. 2014).

We showed in our results in *H. salinarum* that the B-GREAT BFs and OD_Δ_ may be used in combination to characterize differences in growth. In particular, BFs provide an overall metric of growth phenotype significance, and OD_Δ_ quantifies the difference between parent and mutant strain growth across the time course. In general, we find that the use of BFs is a conservative method of finding significantly different growth phenotypes relative to OD_Δ_. For example, there is one case in which OD_Δ_ is significant but the BF is not (e.g., Δ*VNG*1179*C*) (Fig. 3). As such, OD_Δ_ and BF tests provide two tiers of statistical confidence, providing a stringent test to detect phenotypic differences while correcting for variability in the data. By using BF and OD_Δ_ together, an experimental researcher can prioritize strains or conditions to pursue for further study.

B-GREAT recapitulates known biology and discovers previously uncharacterized phenotypes. We confirmed the known growth defect for Δ*trmB* under standard conditions (Fig. 3B–D), which results from its function as a master regulator of metabolic pathways (Schmid et al. 2009; Todor et al. 2013, 2014, 2015). In contrast, the Δ*asnC* oxidative stress phenotype observed previously (Plaisier et al. 2014) was not recapitulated here, likely because the growth defect of this mutant under standard conditions explains the difference in growth during stress (Fig. 3C; Supplemental Fig. S8), which was not corrected for in the previous study (Plaisier et al. 2014). Finally, we identified a previously undiscovered relationship between Δ*sirR* and oxidative damage (Fig. 4). SirR regulates metal uptake transporters at the level of transcription (Kaur et al. 2006), repressing manganese uptake transporters under replete conditions. This regulatory link between metal homeostasis and oxidative stress is well-established in bacterial and eukaryotic organisms (Imlay 2003) but is only beginning to be appreciated in archaeal species (Zhu et al. 2013).

B-GREAT was next applied to population growth data of diverse yeast species under different stress conditions, identifying a previously uncharacterized difference between the growth of *S. cerevisiae* and *S. paradoxus* in response to PQ exposure (Supplemental Fig. S10). Our results also recapitulate the known resistance of *S. paradoxus* to cyclohexamide (Supplemental Fig. S13; Liti et al. 2009). *S. paradoxus* harbors higher levels of ROS (Deregowska et al. 2015), and our results suggest that this may lead to a higher susceptibility to oxidative stress. Many other large-scale population growth studies have been performed to differentiate biological function through population growth phenotypes in the yeast community, and we anticipate that future applications of B-GREAT will highlight additional results from these studies (Warringer et al. 2003; Fernandez-Ricaud et al. 2005).

In future work, B-GREAT will be applied to many problems in testing functional data for significant differential responses to perturbation. Gene expression time-series studies could benefit from this method, where each gene can be tested for differential dynamic profiles between conditions of interest (Bar-Joseph et al. 2012). Population genome-wide association studies (GWAS) are also a potential application of this method to detect the effect of different loci on function responses (Fusi and Listgarten 2016). By adding new covariates, B-GREAT may also be extended to model continuous effects such as dose response (Sekse et al. 2012; Di Veroli et al. 2015; Twarog et al. 2016). By using a time-dependent variance parameter rather than a stationary kernel, B-GREAT may also be extended to model functional data in which heterogeneity between samples is a function of time (Cao et al. 2010). B-GREAT provides a strong foundation to perform and extend the interpretable analysis of the large and growing quantity of dynamic, functional biological data.

## Methods

### *H. salinarum* growth data

Growth of seven TF mutant strains for *H. salinarum*, each deleted in-frame for a TF-encoding gene, and the isogenic Δ*ura3* parent strain was measured (Table 1). Details regarding construction of these mutants were described in prior work (Kaur et al. 2006; Schmid et al. 2009, 2011; Sharma et al. 2012; Plaisier et al. 2014). Cultures were inoculated into complete medium (CM; 250 NaCl, 20 g/L MgSO_4_ · 7H_2_O, 3 g/L sodium citrate, 2 g/L KCl, 10 g/L peptone), grown to stationary phase, and then diluted to OD ∼ 0.05 for growth analysis. OD at 600 nm of 200 independent cultures was measured every 30 min for 48 h using a Bioscreen C (Growth Curves USA). Growth of each strain under each experimental condition was measured in at least biological quadruplicate (from independent colonies) and technical triplicate (independent cultures from the same colony), for a total of 12 replicates. Standard and chronic oxidative stress conditions were tested for all mutants. Standard conditions were defined as 42°C with 225 r.p.m. shaking under ambient light in rich CM medium (Yao and Facciotti 2011). Chronic oxidative stress was induced with 0.333 mM PQ, a redox cycling drug that permeates the cell membrane, that was added at the inoculation of the Bioscreen experiment.

Prior to statistical analysis, OD data were log_2_ transformed and scaled by the estimate of starting OD as follows. Data from growth experiments were grouped by their strain and media composition (e.g., Δ*ura3*, standard growth). This corresponds to the 12 replicates comprising four biological replicates and three technical replicates. Then OD measurements from the first 10 time points within each group were fit with a polynomial regression of degree five. The OD value at time = 0, as estimated by the polynomial regression, was then subtracted from all data points in the group in order to normalize the starting growth levels at zero for all conditions.

### *H. salinarum* data as input to B-GREAT

Input to the GP model corresponds to measurements *Y*_*t*__,*c*,*r*_ for a given time (1 ≤ *t* ≤ *T*), condition (1 ≤ *c* ≤ *C*), and replicate (1 ≤ *r* ≤ *R*). For standard conditions, time points were taken at 4-h increments across a 48-h experiment. This resulted in 12 observations from each replicate. Additionally, growth measurements from both parent strain and each mutant strain were included (*C* = 2). A total of *T* × *R* × *C* = 288 observations was used for training each GP model under standard conditions. For oxidative stress, time points were taken every 6 h, for a total of eight time points for each replicate. The decrease in time samples used in the oxidative stress models was necessary to incorporate the increase in conditions for both standard and oxidative stress growth. Specifically, conditions include growth for both parent and mutant strain under both standard and oxidative stress conditions (*C* = 4). This corresponds to a total of 384 observations for each GP model under oxidative stress.

### Yeast population growth data

Population growth data for 96 yeast strains were collected from a previous study (Liti et al. 2009). One hundred eighty-six conditions are represented in the data set covering various nutrient and stress conditions. For each condition, a minimum of eight replicates for the control strain, BY4741, were available and two replicates of each yeast strain. Measurements were taken every 20 min for 48 h, leading to 144 time points per replicate.

### GP regression of microbial population growth data

GP regression is a probability distribution on arbitrary functions mapping *x* to *f*(*x*) (Rasmussen and Williams 2006). When observations of *f*(*x*) are distorted with IID Gaussian noise, multiple observations of the function are distributed as a multivariate Gaussian

(1) y(x)∼N(μ(x),Σ).

In our application, *x* represents time and *y*(*x*) = log OD(*x*) represents the log-transformed OD measurement at time *t*. A GP model requires specification of a mean function μ(*x*) and kernel function Σ_*i*,*j*_ = κ(*x*_*i*_,*x*_*j*_), which defines the positive definite covariance matrix Σ. In this work, the mean function was set to zero across inputs, μ(*x*) = 0, as is standard (Rasmussen and Williams 2006). For the kernel, we used a RBF with time point–specific independent Gaussian noise:

(2) $$\kappa (x_{i},x_{j})=\sigma_{\text{RBF}}^{2}\cdot \text{exp}\left(\frac{-||x_{i}-x_{j}|{|}^{2}}{\ell^{2}}\right)+\sigma_{\text{nugget}}^{2}\cdot \delta_{x_{i}=x_{j}}.$$

Here, *x*_*i*_ and *x*_*j*_ are two time points; σ_RBF_^2^ is the RBF variance parameter; σ_nugget_^2^ is the Gaussian variance at a single time point *t* (called the nugget); $\delta_{x_{i}=x_{j}}$ is an indicator function, which is equal to one when *x*_*i*_ = *x*_*j*_ and to zero otherwise; and ℓ is the RBF *length scale* parameter, which dictates the smoothness of the function *f*(*x*) through the GP distribution. Kernel function parameters θ = {σ_RBF_^2^, σ_nugget_^2^, ℓ} were optimized by maximizing the likelihood of the data marginalized over the latent function *f*(*x*) with respect to each parameter (Rasmussen and Williams 2006). All GP regression models were built and optimized using the GPy package (version 0.8.8) for Python (http://github.com/SheffieldML/GPy).

#### Other kernels tested

Two other kernels were tested for comparison to RBF kernels, the Matérn and linear kernels. Matérn kernels are defined as

(3) $$k(r)=\sigma^{2}(1+\sqrt{3}r)\text{exp}(-\sqrt{3}r)\;\text{where}\;r=\sqrt{\frac{{\left(x_{i}-x_{j}\right)}^{2}}{\ell^{2}}}.$$

Linear kernels are defined as

(4) $$k(x_{i},x_{j})=\sigma_{i}^{2}x_{i}x_{j}.$$

Model fit for each kernel was assessed with the data likelihood of the optimized GP model and also using the BIC (Neath and Cavanaugh 2012). BIC is calculated as

(5) −2×log(L)+k×log(n),

where *L* is the likelihood of the data, *k* is the number of hyperparameters for each kernel, and *n* is the number of data points.

### GP growth curve metrics

The growth curve metrics μ_max_ and carrying capacity *A* were calculated from the maximum a posteriori (MAP) estimates of either log(OD) or (*d*/*dx*)log(OD) for carrying capacity and μ_max_, respectively. MAP estimates of log(OD) are given by the model in equation 1, by taking the MAP growth level using the fitted model. In order to calculate a MAP estimate of (*d*/*dx*)log(OD), we estimate (*d*/*dx*)log(OD) using GP regression. The RBF kernel is infinitely differentiable, so derivative observations of a GP regression model are also distributed as a GP as follows (Solak et al. 2003):

(6) $$\frac{d}{dx}\text{log}(\text{OD})∼\text{GP}\left(\frac{d}{dx}\mu ,\frac{d}{dx}\Sigma \right),$$

where

(7) $$\frac{d}{dx}\mu =0$$

and

(8) $$\begin{array}{rlr} \frac{d}{dx}\kappa (x_{i},x_{j}) & =\frac{2\cdot \sigma_{\text{RBF}}^{2}}{\ell }\times \left(1-\frac{2\cdot {\left(x_{i}-x_{j}\right)}^{2}}{\ell }\right)\\ & \;\cdot \text{exp}\left(\frac{-||x_{i}-x_{j}|{|}^{2}}{\ell }\right). & \end{array}$$

The GP model of (*d*/*dx*)log(OD) was used to calculate the MAP estimate of (*d*/*dx*)log(OD) as an estimate of μ_max_.

The estimate of AUC was calculated as a metric of the log(OD) distribution as a function of time *t*. The posterior distribution of OD measurements over time is predicted as a MVN, log(OD)(*t*) ∼ *N*(μ(*t*)),Σ(*t*,*t*′)). Predictions were made at 50 evenly spaced time points during the growth curve, and the linear transformation was made of log(OD)(*t*) on the vector *a* = {Δ*t*,Δ*t*,…}, where Δ*t* is the space between predicted time points. This linear transformation is then an approximation of the AUC for the condition, with a normal distribution AUC ∼ *N*(*a*·μ,*a*Σ*a*^*T*^) (Todor et al. 2014).

### Primary growth models

We compared the predictions from the fitted GP regression model to predictions from four primary growth curve models: Gompertz, population logistic, Schnute, and Richards regression (Zwietering et al. 1990). All model parameters were optimized with the curve_fit function of the scipy Python package, which estimates function parameters using damped least squares (Millman and Aivazis 2011). Input data were randomly divided into training (80%) and test (20%) sets for each of the 721 total growth curves in the data set. The MSE of each model fit with respect to the 20% held out test data was calculated as the difference between prediction and test data from models estimated using the training data:

$$\text{MSE}(y,m)=\frac{1}{T}\sum_{t=1}^{T}{\left(y_{t}-m_{t}\right)}^{2},$$

where *y*_*t*_ and *m*_*t*_ correspond to raw data and model predictions at the *t*th time point, respectively. Model prediction *m*_*t*_ was the posterior mean of the fitted GP, and primary growth model predictions were taken from the growth level predicted by the estimated parameters. By use of a one-sided sample *t*-test, MSE for GP regression fit was compared separately to each of Gompertz, population logistic, Schnute, and Richards regression fits. These primary models were selected to compare against the most widely used primary models in modeling microbial population growth (McKellar and Lu 2003). Additionally, the models chosen have been shown to be related to one another through specific constraints on parameters. For example, Gompertz regression can be recovered from the Schnute model with parameters *a* > 0 and *b* = 0 (Zwietering et al. 1990). Therefore, we can observe the improvement of primary model accuracy as we add additional parameters.

#### Gompertz regression

(9) $$y(t)=A\cdot \text{exp}\left[-\text{exp}\left[\frac{\mu_{\max}\cdot e}{A}(\lambda -t)+1\right]\right],$$

where *A* is the carrying capacity, μ_max_ is the maximum growth rate, and λ is lag time (Zwietering et al. 1990).

#### Population logistic regression

(10) $$y(t)=A\cdot {\left[1+\text{exp}\left(\frac{4\cdot \mu_{\max}}{A}(\lambda -t)+2\right)\right]}^{-1},$$

where *A* is the carrying capacity, μ_max_ is the maximum growth rate, and λ is lag time. (Zwietering et al. 1990).

#### Schnute model

(11) $$y(t)=\mu_{\max}\cdot \frac{1-b}{a}\cdot {\left[\frac{1-b\cdot \text{exp}(a\cdot \lambda +1-b-a\cdot t)}{1-b}\right]}^{1/b},$$

where μ_max_ is the maximum growth rate, λ is lag time, and *a*, and *b* are parameters that affect the growth curve shape but do not have direct biological interpretation (Zwietering et al. 1990).

#### Richards model

(12) $$\begin{array}{r} y(t)=A\cdot {\left[1+v\cdot \text{exp}(1+v)\cdot \text{exp}\left(\frac{\mu_{\max}}{A}\cdot (1+v)\cdot \left(1+\frac{1}{v}\right)\cdot (\lambda -t)\right)\right]}^{-1/v} \end{array},$$

where *A* is the carrying capacity, μ_max_ is the maximum growth rate, λ is lag time, and *v* is a parameter that affects the growth curve shape but does not have direct biological interpretation (Zwietering et al. 1990).

#### Testing for significant parameter differences in classical models

Growth parameters μ_max_ and carrying capacity were tested under standard condition and oxidative stress by taking the corresponding parameter estimates for Δ*ura3* and each mutant strain and computing a *t*-test for significant differences between the two populations of parameter estimates.

### Testing for differential growth using BFs

We developed an approximate BF test statistic to quantify possible differences between a pair of growth conditions BF_strain_ (Kass and Raftery 1995; Stephens and Balding 2009). BFs were calculated as the ratio of data likelihoods between an alternative model (*H*_*a*_) and a null model (*H*_0_):

(13) $$\text{BF}=\frac{\;p(Y|H_{a})}{p(Y|H_{0})}.$$

Larger values of the BF indicate a higher relative likelihood under the alternative model and provide evidence for the alternative model representing the data better than the null model.

Specifically, we designed three different BF test statistics to measure differences in population growth across covariates. Under standard conditions, we use BF_strain_, in which the null model *H*_0_ assumes that growth is the same across the parent and mutant strain; the alternative model *H*_*a*_ captures growth between the parent and mutant strain separately. A high BF then suggests that the growth phenotype is different across strains. We designed a second test for differential growth in the presence of oxidative stress, BF_stress_, where the alternative model included an interaction term between genetic effect and oxidative stress. High BF scores under this condition indicate that the mutant strain has a differential growth phenotype relative to the parent strain under oxidative stress. We designed a third test for differential growth across two separate studies, BF_study_, which performs the same test as BF_stress_ but shares statistical strength across batches of growth measurements using a hierarchical GP model.

A FDR for each BF was calculated using an estimate of the null BF distribution, representing BF scores when no significant growth effect between the two conditions is observed. For a single growth experiment, *Y* = {*y*_1_,*y*_2_,…*y*_*T*_} and corresponding time, genetic background, and other covariates *X* = {*x*_1_,*x*_2_,…*x*_*T*_}, each *x*_*t*_ = {time, strain,…}, were randomly assigned a value for *strain* that preserved the original distribution of *strain* values in *X*. One hundred permutations of the data indices following this design were constructed, and a BF score was calculated for each permutation. The distribution of permuted BF scores was used as an estimate of the null distribution of the test statistic, and a BF score that exceeded 80% of permuted scores (corresponding to FDR ≤20%) was selected as significant.

More generally, FDR is calculated using permutations, for a given BF threshold *c*, as follows:

(14) $$\mathrm{FDR}(c)=\frac{|BF_{\mathrm{perm}}>c|}{|BF_{\mathrm{real}}>c|},$$

which approximates the FDR, i.e., the number of false positives over the total number of discoveries, for threshold *c*. In this case, there is a single BF_real_ for 100 permuted BFs, so we multiplied the BF_real_ count by 100 for this computation.

### Differential mutant growth phenotypes

The effects of gene deletion on growth were modeled as experimental effects by extending the input variable *x*, originally representing time, to include perturbations as additional covariates in the GP regression model. The RBF kernel function was extended to handle the additional covariates by using an automatic relevance determination (ARD) prior to induce sparsity on the weighted contribution of each of the *K* covariates (MacKay 1992; Tipping 2001; Rasmussen and Williams 2006; Neal 2012):

(15) $$K(x_{i},x_{j})=\sigma^{2}\cdot \text{exp}\left(\sum_{k=1}^{K}\frac{||x_{i,k}-x_{j,k}||}{\ell_{k}}\right),$$

where each ℓ_*k*_ is the length-scale for the *k*th covariate. These length-scale parameters are then interpretable in terms of quantifying the relative contribution of each of the *k* covariates. Genetic background was incorporated into the model covariates as a Boolean variable *x*_strain_ ∈ {0,1}, where a value of zero indicates parent strain and one indicates mutant strain. For standard growth conditions, *x* has the form

(16) x={time,strain},

whereas the null model contains no strain information: *x* = {time}. The BF then quantified the improvement in data likelihood of the GP regression model, including the strain information versus omitting strain information; when modeling strains separately improved the data likelihood, this indicated that there was differential growth across strains.

### Differential response to stress conditions across strains

Differential growth in response to PQ exposure was tested by extending the covariates to include two additional covariates. The first covariate, mM PQ ∈ {0,1}, represents the presence (1) and absence (0) of PQ stress. The second covariate, mM PQ × strain ∈ {0,1} is an interaction term between mutant strain and stress condition, computed by multiplying the strain covariate with the mM PQ covariate. mM PQ × strain covariate was one only for growth measurements made under oxidative stress for the mutant strain and was zero otherwise. The test for significant growth phenotypes was then made using models including or excluding the mM PQ × strain interaction term. Specifically, the input *x* for the PQ condition had the form

(17) x={time,strain,mMPQ,(mMPQ×strain)}.

The null model, where there is no interaction between strain and stress condition, corresponds to

(18) x={time,strain,mMPQ}.

### Modeling batch effects and testing for differential effects across studies

Growth data for Δ*rosR* under standard conditions and oxidative stress were collected both in this study and in a previous study (Sharma et al. 2012). We modeled the joint growth data from both studies with a hierarchical GP model (Hensman et al. 2013). Under this model, the underlying growth function is modeled with a GP: *g*(*x*) ∼ GP(μ_*g*_,*K*_*g*_). Different batch observations of this function are drawn from a GP with mean equal to *g*(*x*): *f*(*x*) ∼ GP(*g*(*x*),*K*_*f*_).

Growth data for Δ*rosR* and the parent strain were modeled by replicate functions *f*_1_ and *f*_2_, representing data from our study and the previous study, respectively. The GP models for *f*_1_, *f*_2_, and *g* all follow the design in equation 17. BF scores in both cases were calculated as the difference in log likelihood for GP models accounting for strain variation interacting with oxidative stress (*H*_*A*_; equation 17) and those that do not interact with oxidative stress (*H*_0_; equation 18). The BF permutation was performed as described above.

### Computing differences between population growth across time series (OD_Δ_)

The difference between mutant and parent strain functions across time points were defined by the variable OD_Δ_. The variable OD_Δ_ is the difference in mutant strain growth and parent strain growth at each time point *t*_*k*_. OD_Δ_ was calculated using the noiseless latent mean function for population growth rather than the noisy observations. In other words, we use the latent function *f*: log(OD) = *f*(*t*) + ε, where *f*(*t*) is the smooth underlying growth function and ε represents random noise. OD_Δ_ at a specific time point *t*_*k*_ is then the difference between the growth of the mutant strain *f*_*m*_ and the parent strain *f*_*p*_:

(19) $$f_{m}(t_{k})-f_{p}(t_{k}),$$

where *f*_*m*_ and *f*_*p*_ are estimated from the posterior distribution of the trained GP model. Finally, OD_Δ_ also corrects for differences between parent and mutant strain at the start of the experiment, *t*_0_, by subtracting their respective growth levels at that time point:

(20) $$OD_{\Delta }(t_{k})=(\;f_{m}(t_{k})-f_{p}(t_{k}))-(\;f_{m}(t_{0})-f_{p}(t_{0})).$$

The four variables needed to calculate OD_Δ_—i.e., *f*_*k*_ = {*f*_*m*_(*t*_*k*_), *f*_*m*_(*t*_0_), *f*_*p*_(*t*_*k*_), and *f*_*p*_(*t*_0_)}—are defined by a joint MVN distribution predicted by the fitted GP:

(21) $$\begin{array}{rlr} f_{k} & ={\left[\;f_{m}(t_{k}),f_{m}(t_{0}),f_{p}(t_{k}),f_{p}(t_{0})\right]}^{T}\\ & \;∼N({\left[\mu_{m}(t_{k}),\mu_{m}(t_{0}),\mu_{p}(t_{k}),\mu_{p}(t_{0})\right]}^{T},\Sigma_{k}). & \end{array}$$

OD_Δ_ is then a linear transformation of these variables, OD_Δ_ = *a*·*f*_*k*_, where *a* is the column vector *a* = [1,−1,−1,1] (*a*:1 × 4). Parameter OD_Δ_ is then distributed as a univariate normal distribution, OD_Δ_ ∼ *N*(*a* · μ_*k*_, *a*Σ_*k*_*a*^*T*^ = *N*(μ(OD_Δ_), σ^2^(OD_Δ_)). Credible intervals of OD_Δ_ as defined by its normal distribution were calculated to determine whether OD_Δ_ = 0 lies within the 95% credible region. If zero was not in this region, the difference between parent and mutant strain was considered significant at this time point.

### Applying B-GREAT to yeast population growth data

Population growth data for 96 yeast strains under cycloheximide exposure and PQ exposure were modeled using the stress interaction test of B-GREAT (equation 17), where mM PQ was substituted for mM cycloheximide where appropriate. The strain BY4741 was used as the control strain, and growth in yeast extract peptone dextrose (YPD) was used as the control growth condition. BF calculation and permutations of BF scores for each strain were performed as described above.

#### Testing for enrichment in significant strains

Significance for enrichment of *S. paradoxus* strains as having significant growth phenotypes was tested using a hypergeometric distribution, using the hypergeom package from scipy.

## Data access

All code and data from this study have been submitted to https://github.com/ptonner/gp_growth_phenotype and are available in the Supplemental Methods archive. Raw growth data for *H. salinarum* used in this study are available in Supplemental Table S1.

## Supplementary Material

Supplemental Material
