# Supplementary material for: Detecting differential growth of microbial populations with Gaussian process regression
Source: Genome Res. 2017 Feb;27(2):320–33. doi: 10.1101/gr.210286.116 (PMC5287237; doi:10.1101/gr.210286.116)
Supplement: Supplemental Material [file supp_27_2_320__index.html]

Detecting differential growth of microbial populations with Gaussian process regression — Supplemental Material 

# Detecting differential growth of microbial populations with Gaussian process regression

## Supplemental Material

- Supplemental\_Methods.zip
- Supplemental\_Table\_S1.xls
- Supplemental\_Table\_S2.xlsx
- Supplemental\_Material.pdf
