# Supplementary material for: Detecting differential growth of microbial populations with Gaussian process regression
Source: Genome Res. 2017 Feb;27(2):320–33. doi: 10.1101/gr.210286.116 (PMC5287237; doi:10.1101/gr.210286.116)
Supplement: Supplemental Material [file supp_gr.210286.116_Supplemental_Material.pdf]

Supplemental Information for: Detecting differential growth of microbial populations with Gaussian process regression

Peter D Tonner[1,2], Cynthia L Darnell[2], Barbara E Engelhardt[3], Amy K Schmid[1,2]

1 Program in Computational Biology and Bioinformatics, Duke University, Durham, NC 27708, USA

2 Biology Department, Duke University, Durham, NC 27708, USA

3 Computer Science Department, Center for Statistics and Machine Learning, Princeton University, Princeton, NJ 08544, USA

Corresponding Authors: bee@princeton.edu, amy.schmid@duke.edu

Table S1. Growth data for *H. salinarum* TF mutants. Raw OD measurements of each TF mutant strain and $\Delta ura3$ parent strain under standard conditions and oxidative stress. Each row corresponds to a different replicate, containing information on experiment conditions and OD measurements at each time point.

Table S2. Significance of differences between $\Delta ura3$ and each TF mutant in parameters estimated by primary models. The table lists p-values resulting from t-tests between $\Delta ura3$ and each mutant for growth rate and carrying capacity estimated by Gompertz regression. Data for standard and paraquat conditions are given on separate tabs of the excel file.

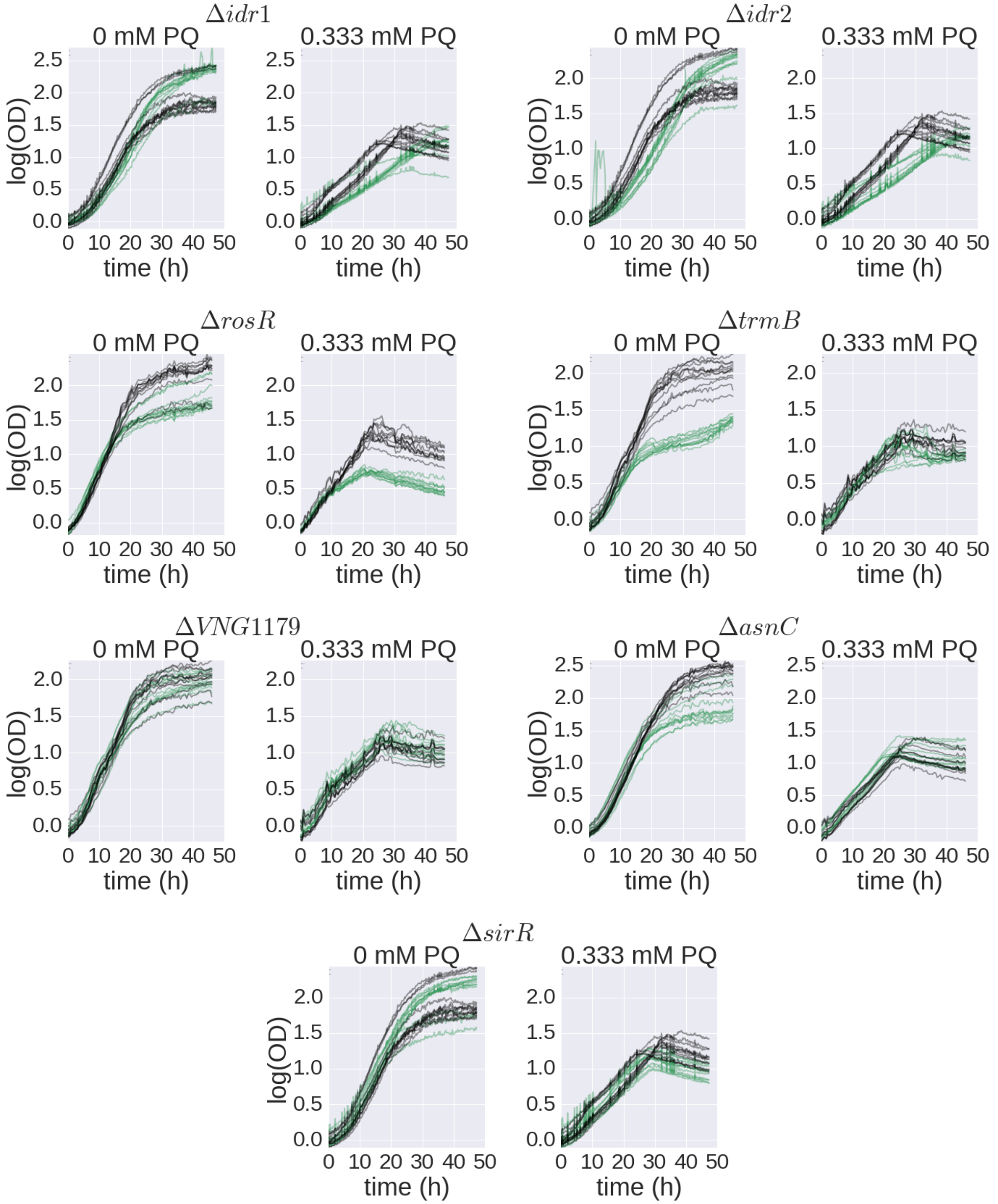

Figure S1. Population growth data of *H. salinarum* under standard and oxidative stress conditions. Optical density measurements of $\Delta ura3$ parent strain (black) and TF mutants (green) under standard conditions [0 mM paraquat (PQ), left] and chronic oxidative stress (0.333 mM PQ, right). Each line represents time series growth data from one replicate sample. Each subpanel represents the growth data for a different mutant.

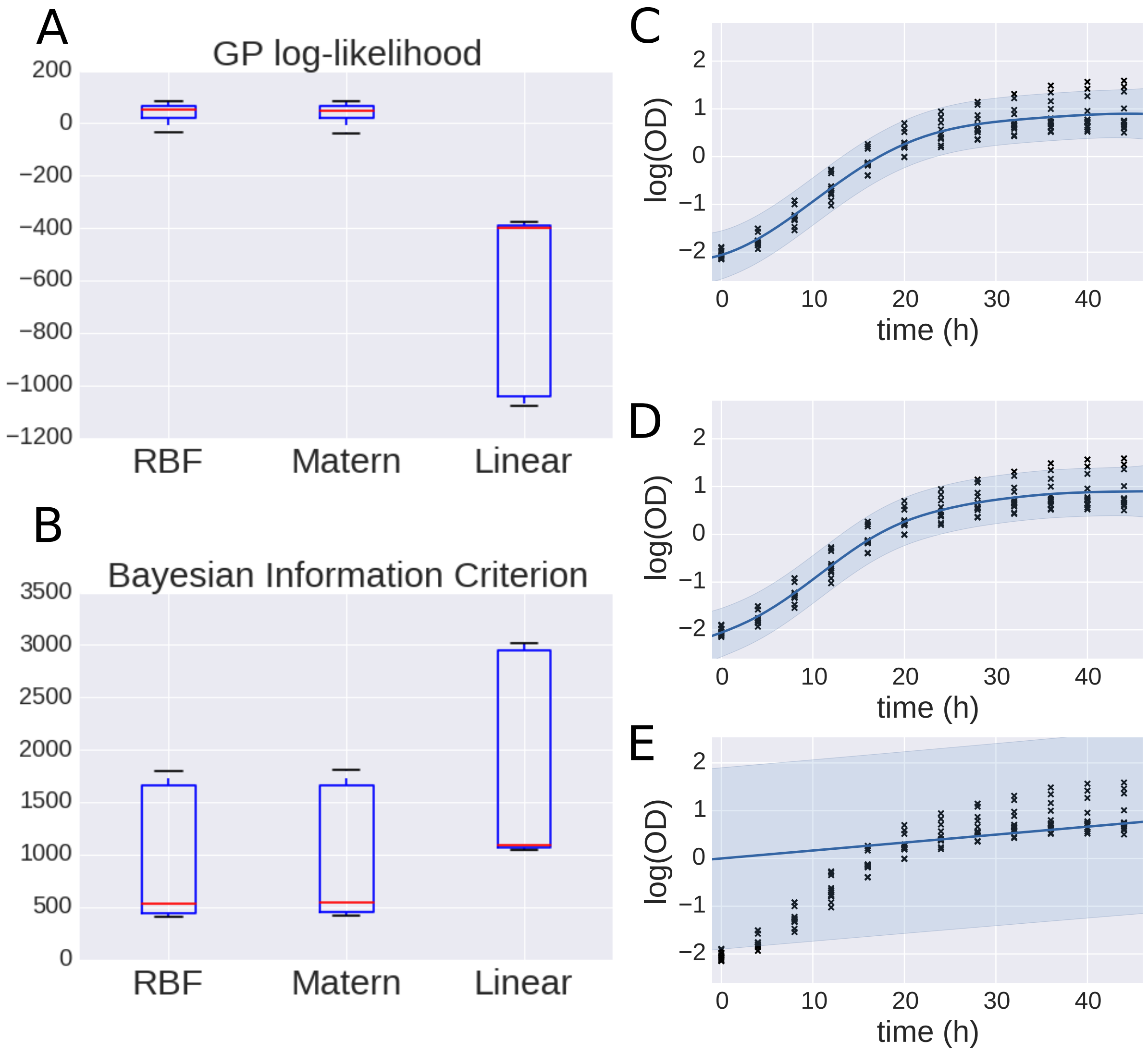

Figure S2. Comparison of kernel fits to microbial population growth data. (A) Distribution of log-likelihood scores of fitted GP models for RBF, Matern, and linear kernels. GP models with each kernel were fit to data as in Fig. 3. (B) Bayesian information criterion (BIC) of GP model for each kernel. (C) Example model fit with RBF kernel. (D) Example model fit with Matern kernel. (D) Example model fit with linear kernel.

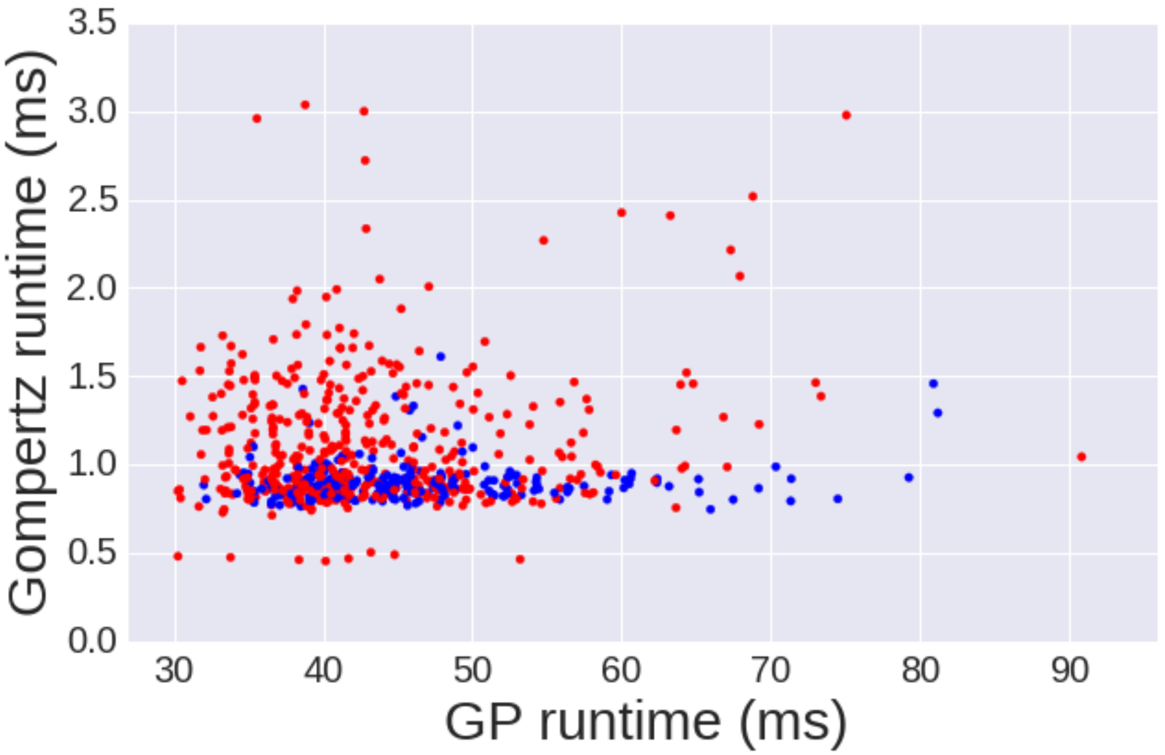

Figure S3. Runtime comparison between GP regression and Gompertz regression. Parameter estimation time for fitting individual growth curve replicates under standard growth conditions (blue points) and oxidative stress conditions (red points) are shown.

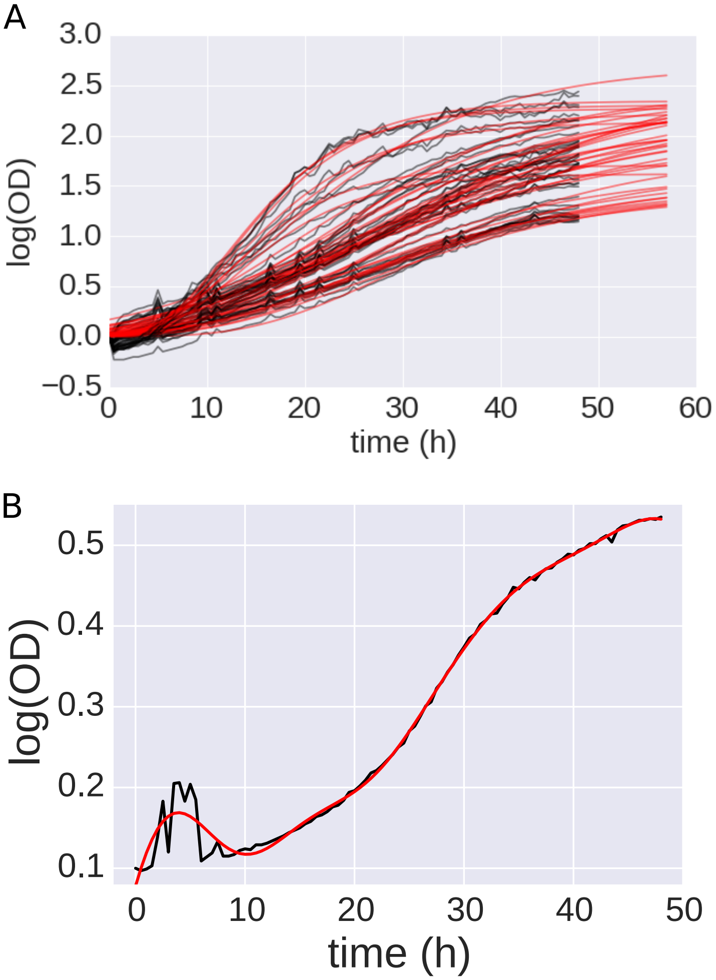

Figure S4. Comparison of GP and Gompertz model fit. (A) Population growth data with largest difference in carrying capacity estimates between GP and Gompertz regression. Optical density measurements (black) and Gompertz regression model fits (red). Gompertz regression assumes that growth proceeds beyond the sampled time frame, as evidenced by the continued increase in model prediction after 48 hours. (B) Population growth data with largest difference in μ_{max} estimates between GP and Gompertz regression. Optical density measurement (black) and GP regression estimate (red) of the single largest outlier in the estimates of μ_{max} between GP regression and Gompertz regression. The large difference in μ_{max} occurs because of a drastic shift in OD measurements early in the growth curve, likely due to instrument error.

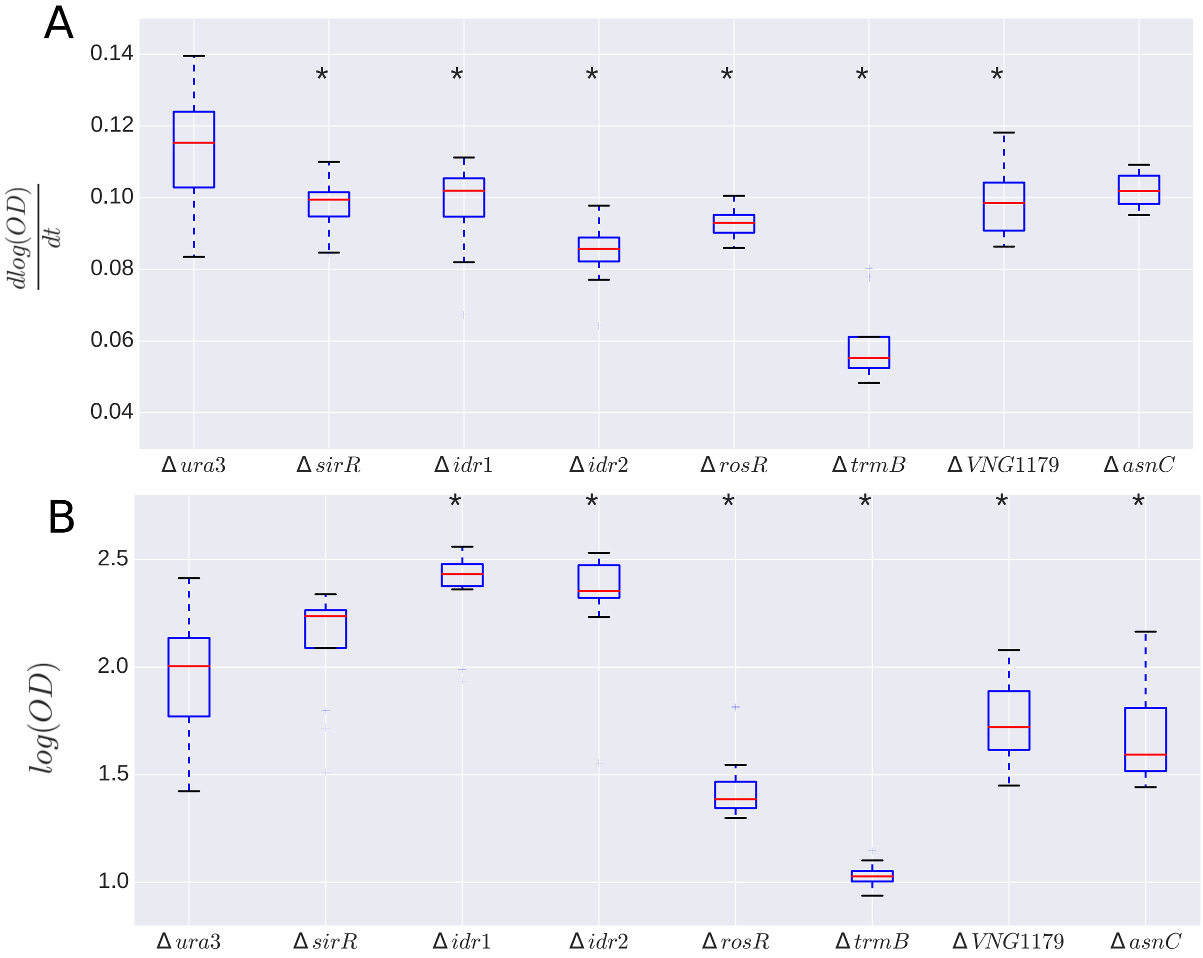

Figure S5. Testing classical growth parameters under standard conditions. (A) Distribution of μ_{max} estimates for each strain under standard conditions. (B) Distribution of carrying capacity estimates for each strain under standard conditions. Asterisks indicate significant difference between $\Delta ura3$ estimates and mutant strain estimates according to p-value score ($p < 0.01$). All p-values for these t-tests are listed in Supplemental Table S2.

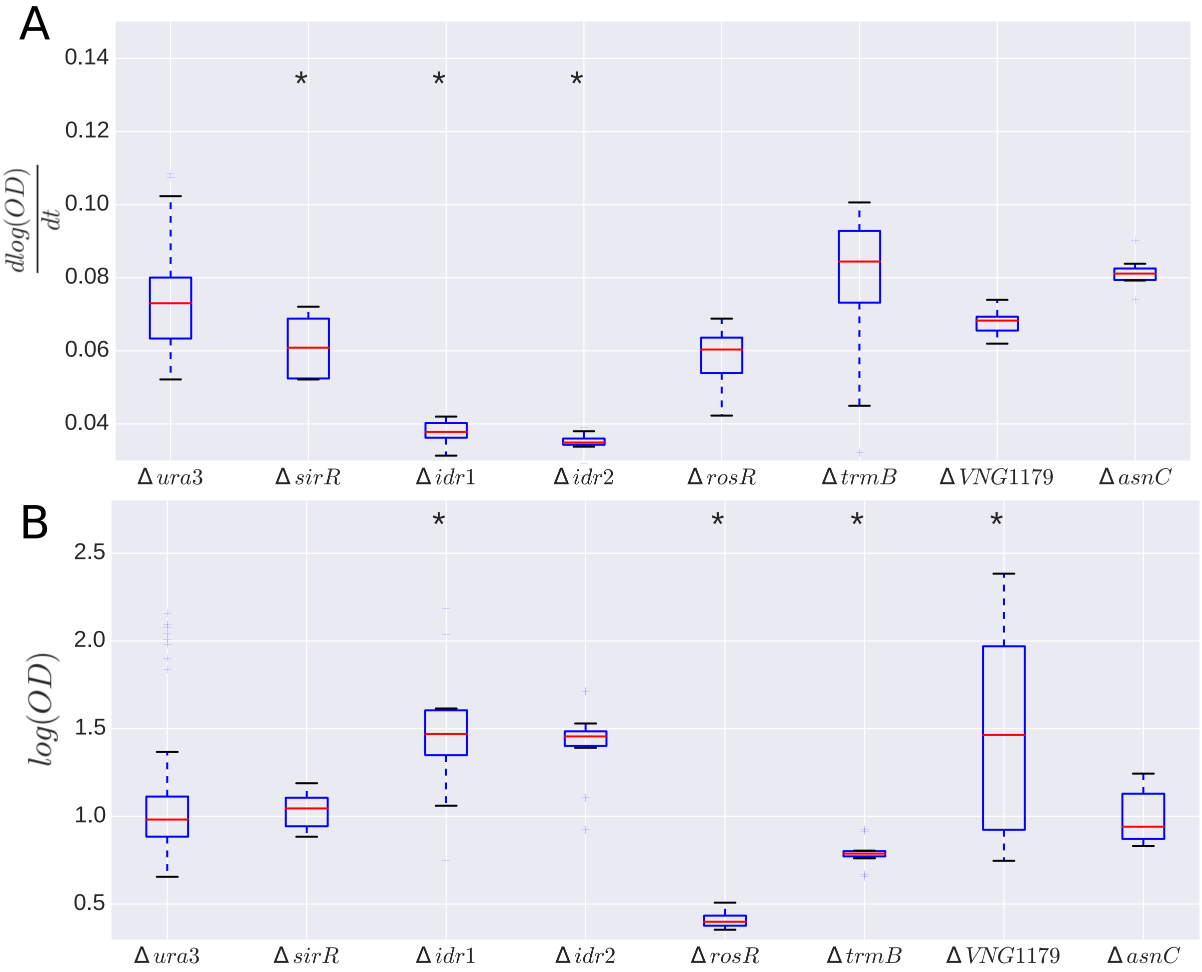

Figure S6. Testing classical growth parameters under oxidative stress. (A), (B), and asterisks as in Supplemental Fig. S5. Parameter estimates are from oxidative stress conditions. All p-values for these t-tests are listed in Supplemental Table S2.

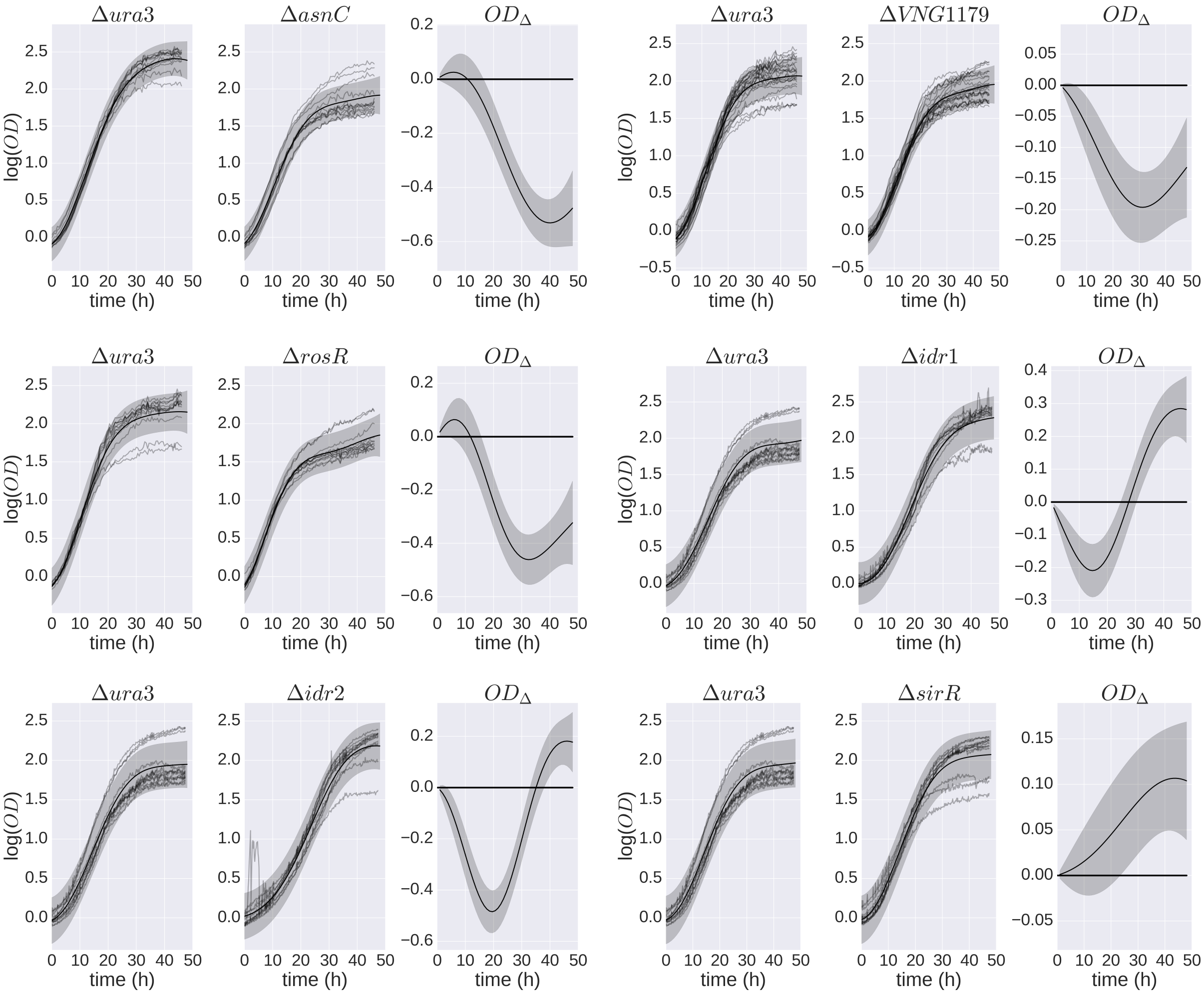

Figure S7. Standard growth phenotypes as identified by GP regression. Growth phenotypes for $\Delta asnC$, $\Delta VNG1179$, $\Delta rosR$, $\Delta idr1$, $\Delta idr2$, and $\Delta sirR$ under standard conditions. Raw growth data is shown as grey lines. Solid lines indicate posterior mean and shaded regions depict posterior variance of growth phenotypes for each strain as predicted by GP regression. Within each subpanel, left plots exhibit growth data for the $\Delta ura3$ parent strain, middle plots show that for mutant strains, and right plots depict posterior growth difference estimates between mutant and parent strains.

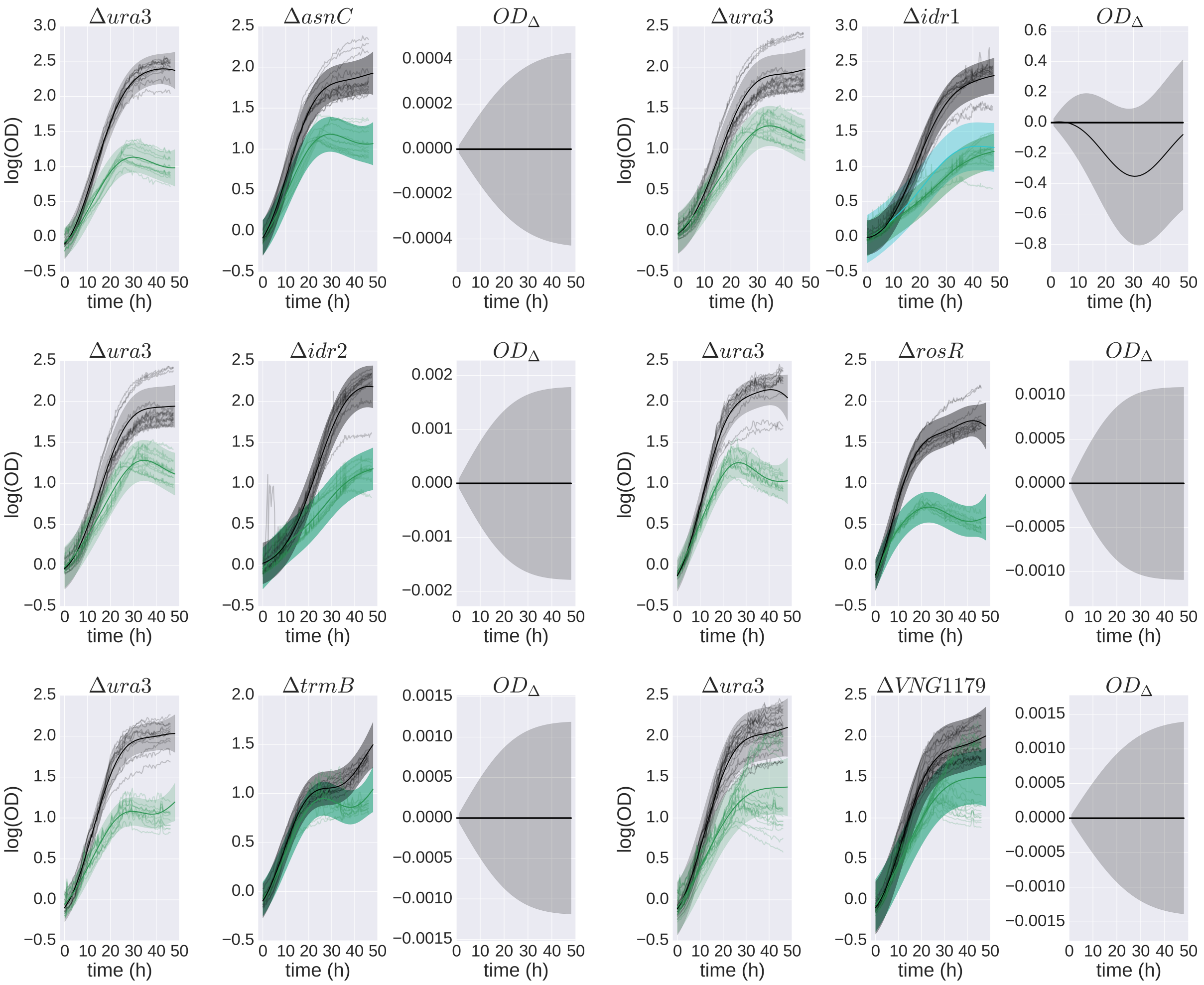

Figure S8. Oxidative stress growth phenotypes as identified by GP regression. Subpanels show growth phenotypes for the following strains under oxidative stress (from left to right): $\Delta asnC$, $\Delta idr1$, $\Delta idr2$, $\Delta rosR$, $\Delta trmB$, and $\Delta VNG1179$. Growth data is shown for standard growth (grey lines) and oxidative stress (light green lines). GP predictions of growth are shown as solid line and shaded regions indicating posterior mean and variance of growth phenotypes, respectively. GP prediction colors match the growth data, and blue predictions correspond to the absence of interaction between strain and stress (mM PQ $\times$ strain $= 0$). Blue regions are not visible in cases where blue and green regions overlap. Within each subpanel, left plots display growth data for the $\Delta ura3$ parent strain, middle plots display that for each of the mutant strains, and right plots depict posterior growth difference estimates between mutant and parent strain (OD_Δ).

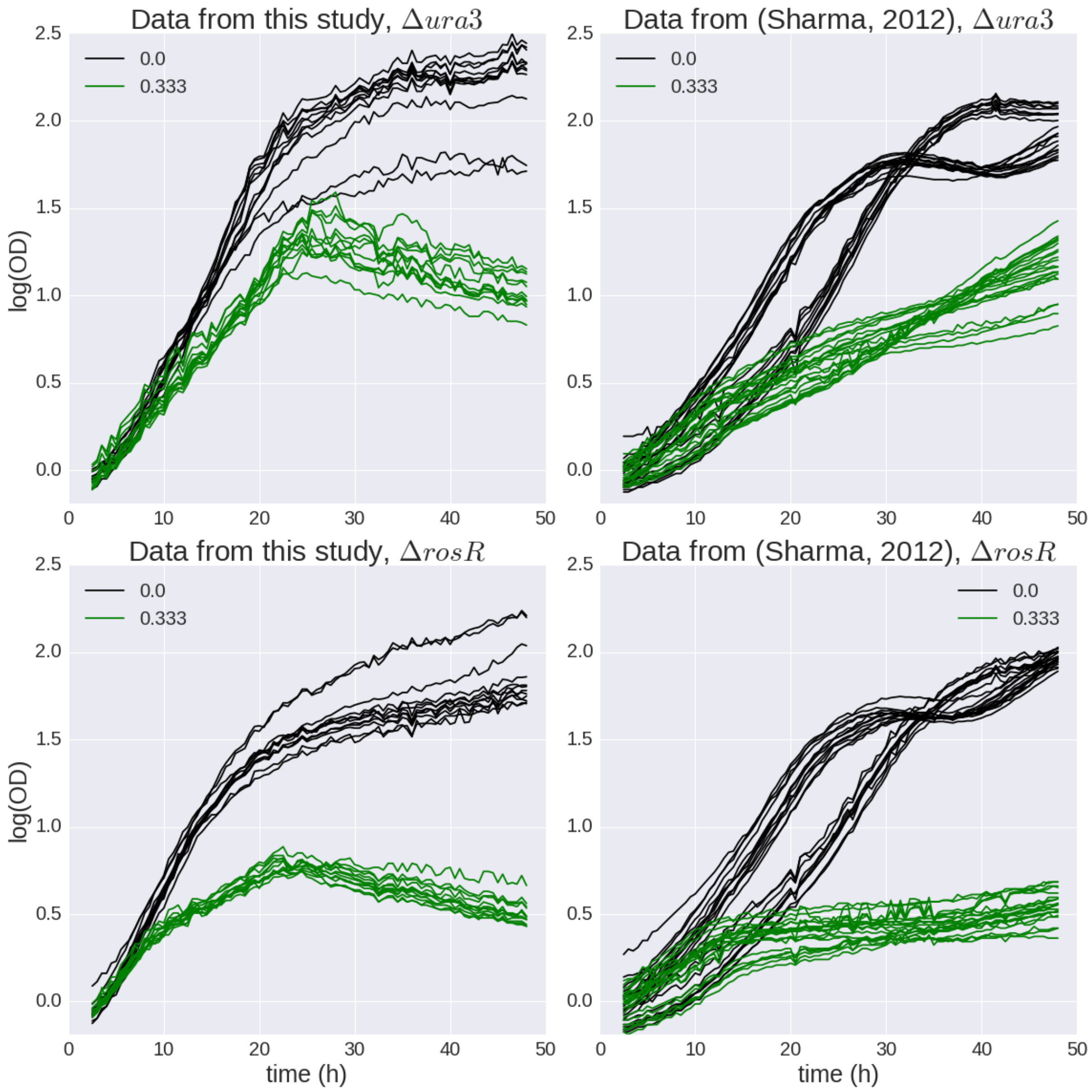

Figure S9. Data used for the batch effects analysis of $\Delta rosR$. Growth data of $\Delta rosR$ and $\Delta ura3$ in this study (left) compared to that from a previous study (Sharma et al. 2012). Top row is measurements from $\Delta ura3$, and bottom row is from $\Delta rosR$. Growth data were measured with either 0 mM PQ (black) or 0.333 mM PQ (green).

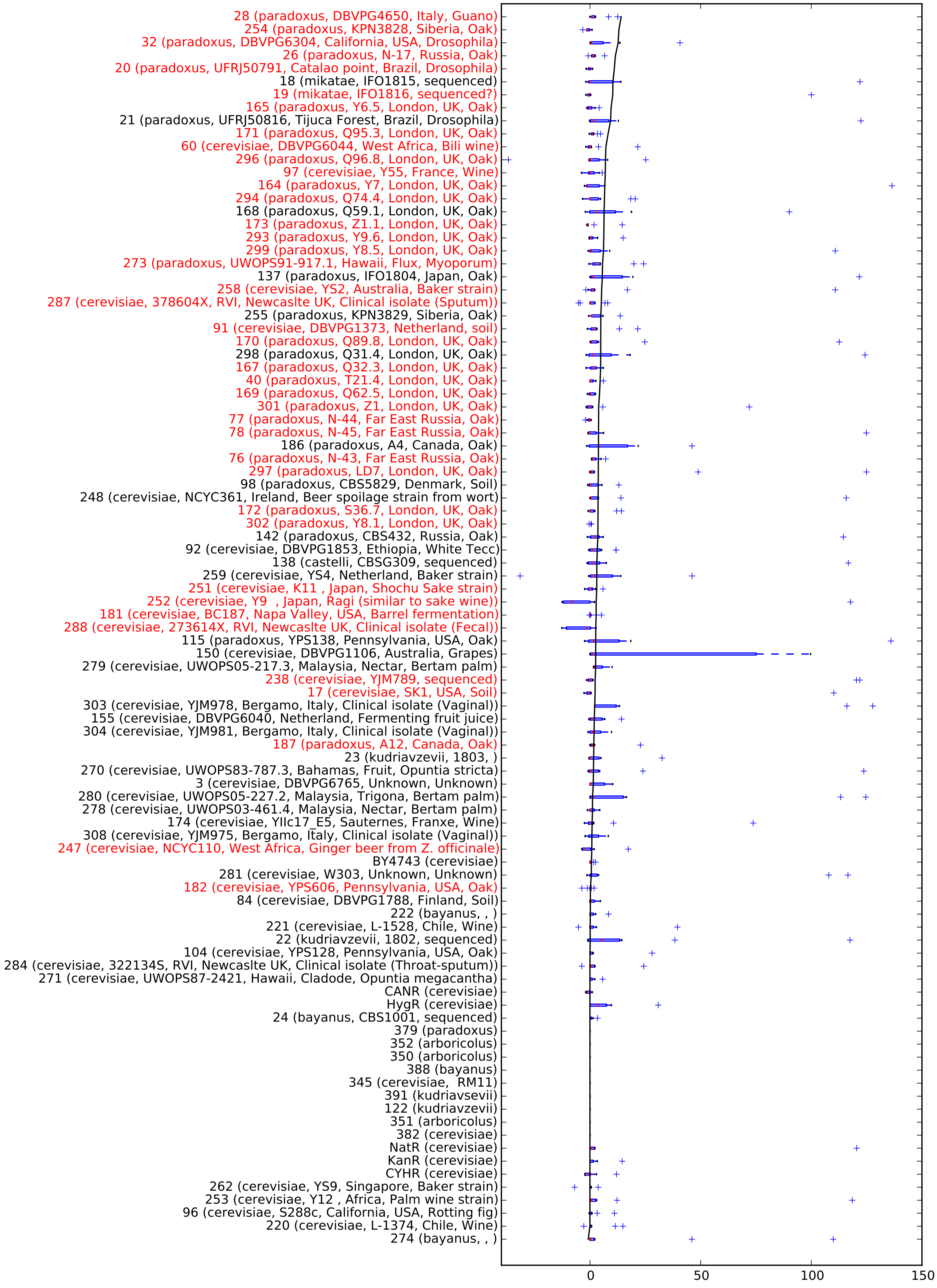

Figure S10. B-GREAT identifies significant growth phenotypes in yeast strains. Bayes factor scores (black line) and permuted BF scores (boxplots) for each yeast strain are shown for differential growth vs. the control strain (BY4741) under paraquat. Strains written in red are significant at an FDR of 20%.

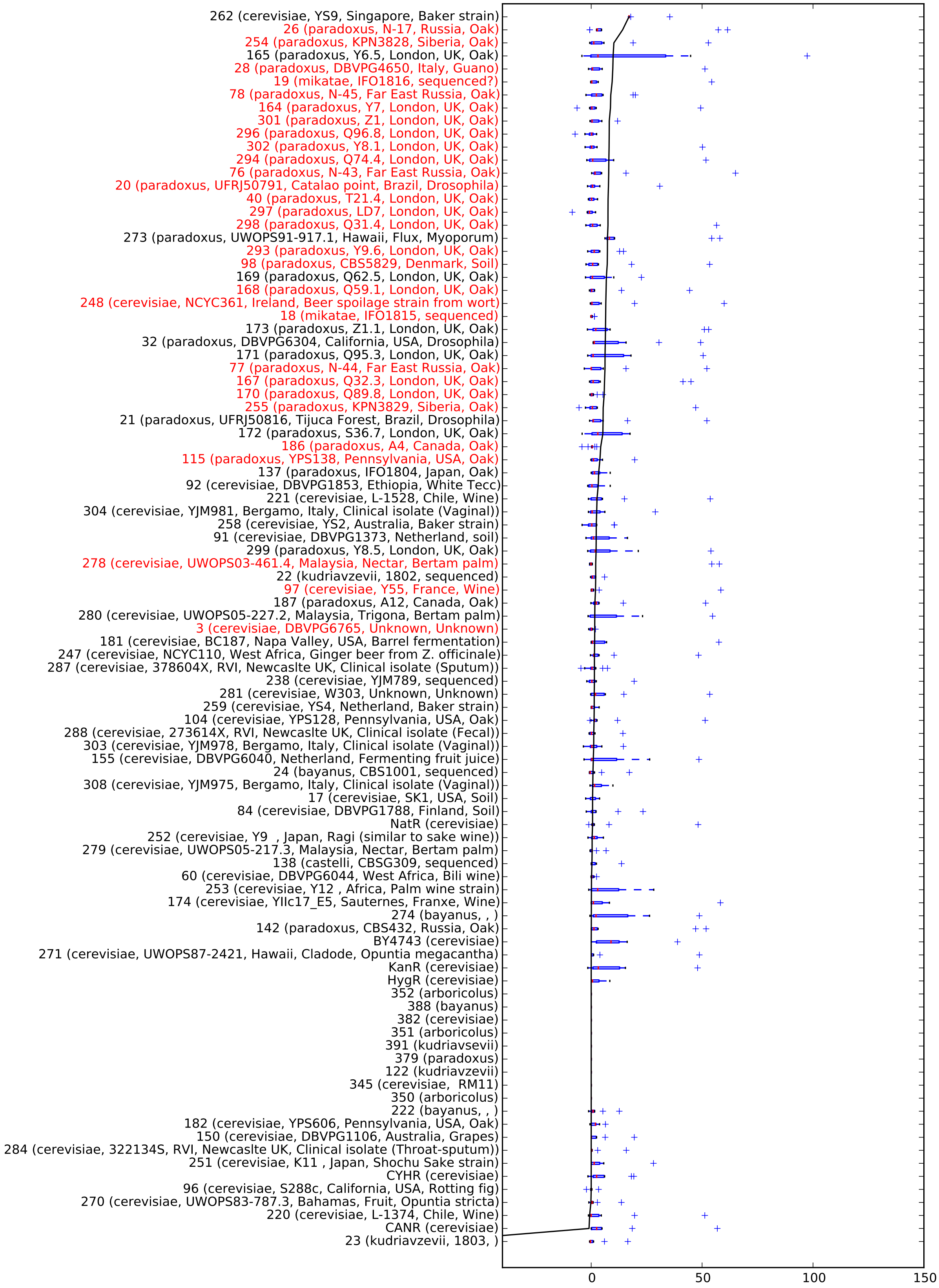

Figure S11. Significant growth phenotypes for yeast strains under cycloheximide stress. Lines, boxplots and strain names as in Supplemental Fig. S10.

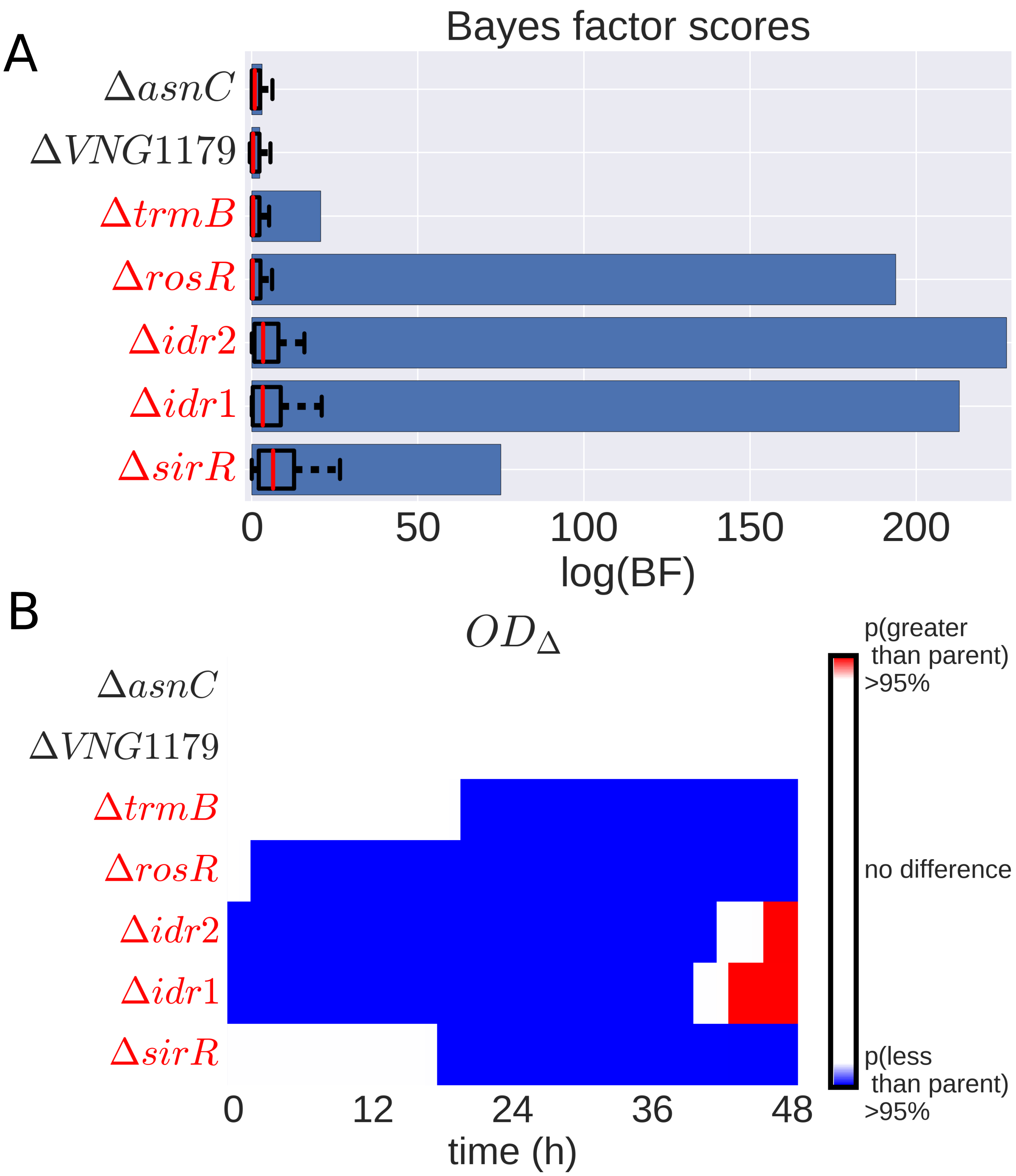

Figure S12. Computing significant growth phenotypes without an interaction term for paraquat stress. (A) Bayes factor scores for each mutant strain under oxdiative stress with no interaction term between strain and stress. Significance of growth phenotype for each mutant strain under oxidative stress was determined using the model of eq. 4 vs. the model of eq. 2. Blue bars represent BF scores and box-whisker plots depict permuted BFs. (B) OD_{Δ} of each strain using the same model as in (A). Colors as in main text Fig. 3.

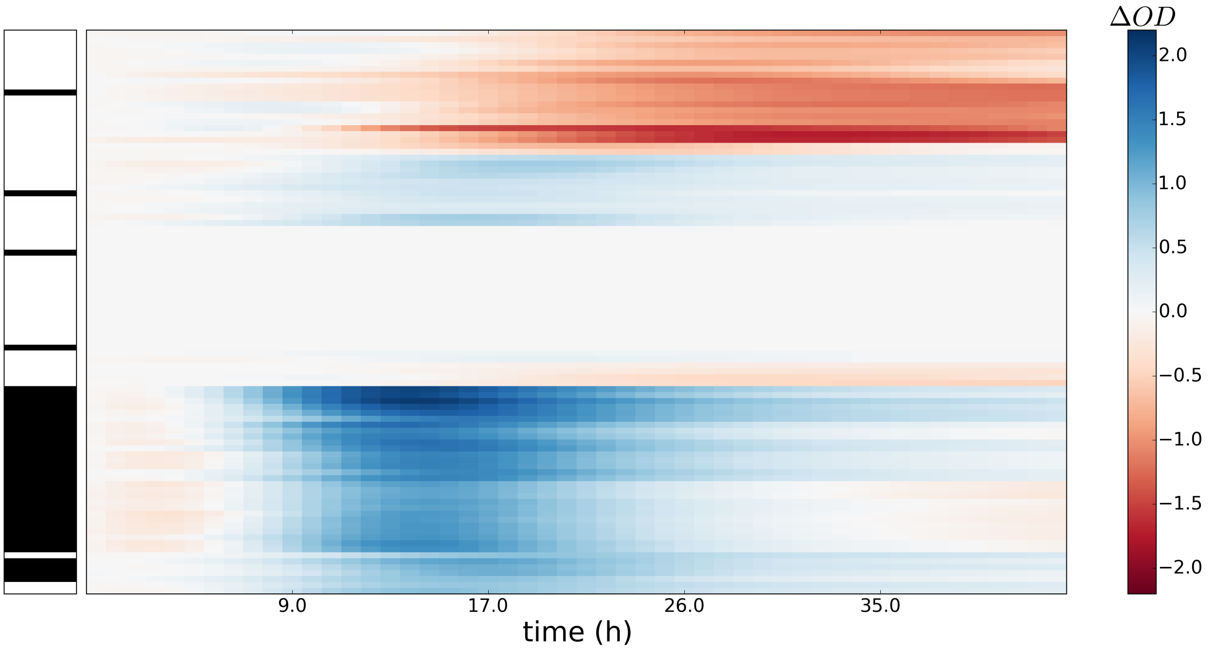

Figure S13. B-GREAT identifies significant growth phenotypes in yeast strains in response to cycloheximide. OD_{Δ} of all yeast strains under cycloheximide exposure. Left column corresponds to *S. cerevisiae* (white) or *S. paradoxus* (black) strains. Center column represents magnitude of calculated OD_{Δ} over time for each strain.
